# CD24, CD44 and EpCAM enrich for tumour-initiating cells in a newly established patient-derived xenograft of nasopharyngeal carcinoma

**DOI:** 10.1038/s41598-017-12045-8

**Published:** 2017-09-28

**Authors:** Susan Ling Ling Hoe, Lu Ping Tan, Norazlin Abdul Aziz, Kitson Liew, Sin-Yeang Teow, Fazlyn Reeny Abdul Razak, Yoon Ming Chin, Nurul Ashikin Mohamed Shahrehan, Tai Lin Chu, Noor Kaslina Mohd Kornain, Suat-Cheng Peh, Cheng Eng Koay, Kwok-Wai Lo, Munirah Ahmad, Ching-Ching Ng, Alan Soo-Beng Khoo

**Affiliations:** 1 Molecular Pathology Unit, Cancer Research Centre, Institute for Medical Research, 50588 Kuala, Lumpur Malaysia; 2 Institute of Biological Sciences, Faculty of Science, University of Malaya, 50603 Kuala, Lumpur Malaysia; 3 Sunway Institute for Healthcare Development, Sunway University, 47500 Bandar Sunway, Selangor Malaysia; 4 Department of Pathology, Faculty of Medicine, Universiti Teknologi MARA (UiTM), 47000 Sungai Buloh, Selangor Malaysia; 5 Gleneagles Hospital (Kuala Lumpur) Sdn. Bhd., Jalan Ampang, 50450 Kuala, Lumpur Malaysia; 6 Li Ka Shing Institute of Health Science, The Chinese University of Hong Kong, Hong Kong, Hong Kong SAR

## Abstract

Subpopulations of nasopharyngeal carcinoma (NPC) contain cells with differential tumourigenic properties. Our study evaluates the tumourigenic potential of CD24, CD44, EpCAM and combination of EpCAM/CD44 cells in NPC. CD44br and EpCAMbr cells enriched for higher S-phase cell content, faster-growing tumourigenic cells leading to tumours with larger volume and higher mitotic figures. Although CD44br and EpCAMbr cells significantly enriched for tumour-initiating cells (TICs), all cells could retain self-renewal property for at least four generations. Compared to CD44 marker alone, EpCAM/CD44dbr marker did not enhance for cells with faster-growing ability or higher TIC frequency. Cells expressing high CD44 or EpCAM had lower KLF4 and p21 in NPC subpopulations. KLF4-overexpressed EpCAMbr cells had slower growth while Kenpaullone inhibition of *KLF4* transcription increased *in vitro* cell proliferation. Compared to non-NPC, NPC specimens had increased expression of *EPCAM*, of which tumours from advanced stage of NPC had higher expression. Together, our study provides evidence that EpCAM is a potentially important marker in NPC.

## Introduction

Nasopharyngeal carcinoma (NPC) is a type of head and neck cancer predominantly found in the southern Chinese population, several indigenous groups of the Southeast Asia, Amazigh- and Arabic-speaking populations of North Africa, and the Inuits of North America and Greenland^1^. It is infamously associated with high dietary intake of salted and/or preserved food, exposure to tobacco and formaldehyde as well as Epstein-Barr virus (EBV) infection^1^. Although it is a highly radiosensitive malignancy, its initial nonspecific clinical presentations such as nasal blockage, blood-tainted sputum, ringing in the ears and mild hearing loss are the main obstacles to early diagnosis^2,3^ and the 5-year overall survival rate for NPC patients can reduce remarkably in late-stage of the disease^4^.

Studies on NPC tumour biology such as growth, stemness, invasion, metastasis, therapy resistance and EBV presence have been widely investigated mainly using NPC cell lines. Stem-like cells from NPC cell lines were commonly enriched by using functional assays such as the side population (SP) assay^5–7^, spheroid assay^8^ and ALDH assay^9^. The SP cells in frequently used CNE-2 and HK1 cell lines were found to be more tumour-inducing in nude mice than bulk cells^6,7^. Cells from another NPC cell line, C666-1 which grew as free-floating spheres, displayed stemness characteristics such as a propensity for tumour formation, contained higher expression levels of pluripotent genes *SOX2* and *KLF4* as well as metastasis-associated gene *CD44* than the normally adherent ones, and had higher tumourigenic potential *in vivo* ^8^. ALDH1 positive cells from 5-8F and CNE-2 cell lines induced tumour growth, contained higher percentage of SP subpopulation and had higher levels of *OCT4*, *BMI1*, *KLF4* and *SOX2* transcripts than ALDH1 negative cells^9^.

As cancer cells from patient samples are made up of heterogeneous cell types potentially with different tumorigenic ability, the use of surface markers have aided in the isolation of these cells directly from clinical samples for the study of their roles in tumourigenesis^10,11^. These markers which are largely associated with tumour growth, metastasis and survival are commonly referred to as cancer stem cell (CSC) markers. There is a dearth of such studies using clinical samples in NPC due to sample size limitation as surgery is not the mainstay treatment modality^12,13^.

Based on the latest reviews on CSC markers in NPC cell lines, CD44, an extracellular receptor for hyaluronan, seems to be the most widely studied marker with roles ranging from tumour initiation, cell proliferation and differentiation to 5-fluorouracil treatment resistance^14,15^. In breast and rat mammary carcinomas, CD24 is known as a marker for metastasis due to its binding to P-selectin which facilitated the passage of tumour cells in the bloodstream during metastasis^16,17^. The absence or low expression of CD24 is synonymous with identifying breast CSCs as was first highlighted by Al-Hajj *et al*.^18^. EpCAM (CD326) or previously known as ESA (epithelial specific antigen) functions as an epithelial-specific intercellular cell adhesion molecule with additional involvement in cellular signalling, cell migration, proliferation and differentiation^19^. It identified cancer cells with tumourigenic and stem-like properties in combination with CD24, CD44 and/or CD166 in breast, lung and gastric carcinomas^18,20,21^. Not much is known, however, regarding EpCAM in NPC.

To the best of our knowledge, the roles of CD24, CD44 and EpCAM in NPC tumourigenesis have not been evaluated within the same cell populations in cell lines or patient-derived xenografts (PDXs). Due to its close resemblance to clinical samples, early-passage PDXs have been advocated to be used as study models for tumour heterogeneity, CSCs and therapy-related studies, instead of cell lines^22–25^. However, most NPC biological studies were performed on NPC cell lines which have been in passage for many generations^8,26,27^ or primary culture derived xenograft^28,29^, some with questionable authenticity and/or origin as were reported for CNE-1, CNE-2 and HONE-1^30,31^. Most importantly, the gold standard for evaluation of CSCs^32,33^ i.e. *in vivo* serial transplantation assay was not used to thoroughly assess self-renewal ability in aforementioned studies on NPC stem-like cells.

In the present study, we evaluated the expression of CD24, CD44 and EpCAM in a set of NPC samples comprising of two cell lines (HK1 and C666-1) and two early-passage PDXs (xeno-284 and xeno-B110) by flow cytometry analysis. Subsequently, CD24, CD44, EpCAM and EpCAM/CD44 marker-selected subpopulations were isolated from C666-1 and xeno-B110. These cells were characterized for tumour initiation, growth ability and tumour-initiating cell (TIC) frequency. In addition, selected cells were examined for self-renewal by serial-transplantation for four generations, gene and protein expressions related to stemness, pluripotency, proliferation and cell cycle. Finally, proliferation-related activity of KLF4 was examined in xeno-B110, and expression of selected mRNA and proteins were assessed in NPC specimens.

## Results

### NPC cell lines and PDXs display variable expression of common surface markers

**Figure 1:**
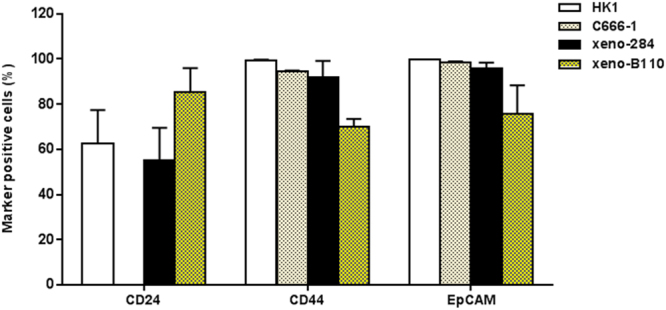
Expression of common surface markers in NPC cell lines and NPC xenografts. Percentage of marker positive cells from the cell lines were counted from the total number of single, viable cells. As for the xenografts, the denominator was total number of single, viable, non-mouse cells. Results, mean ± SD of 3 flow cytometry experiment replicates.

As CD24, CD44 and EpCAM were frequently used to isolate tumourigenic cells^18,21,25,34,35^, their expression levels were assessed in NPC cell lines (HK1 and C666-1 cell lines) and early-passage PDXs (xeno-284 and xeno-B110) by flow cytometry (Fig. 1). Xeno-284 and xeno-B110 are two NPC PDXs newly established in our lab. Prior to use, HK1 and C666-1 cells were authenticated by STR profiling and found to be identical and closely related, respectively, to the ones used by NPC researchers^30^ (Supplementary Table S1). Periodical tests showed that both cell lines were mycoplasma-free. STR data also verified that xeno-284 and xeno-B110 show a high concordance to the original NPC patients’ blood samples and are different from known NPC PDXs such as xeno-666, C15 or C17 (Supplementary Table S1). EBV status in in xeno-B110 and xeno-284 was verified by EBER-ISH method (Supplementary Fig. S1).

CD24 was highest in xeno-B110 (85.37 ± 10.51% positive cells), moderately expressed in xeno-284 and HK1 (55.33 ± 14.17% and 62.77 ± 14.63%, respectively), and extremely low to absent (0.00 ± 0.06%) in C666-1 (Fig. 1). Absence of CD24 expression was also observed in C666-1 cells passaged *in vivo* (“xeno-C666-1”) (Supplementary Fig. S2). CD44 was moderate to highly expressed in all samples with the lowest level in xeno-B110 (70.15 ± 3.23%) and the highest in HK1 (99.47 ± 0.15%) (Fig. 1). More than 95% EpCAM positivity was detected in all samples except for xeno-B110 (75.79 ± 12.45%) (Fig. 1).

NPC is prevalently EBV positive, hence subsequent experiments were performed using EBV positive C666-1 and xeno-B110 samples derived from primary NPC specimens, as opposed to EBV negative HK1 and xeno-284 which were established from recurrent NPC specimens. Also, as the negative subpopulations of CD24, CD44, EpCAM and EpCAM/CD44 were scarce in C666-1 and xeno-B110, bright and dim phenotypes of each marker were studied for their biological properties (Supplementary Fig. S3). Marker bright and dim phenotypes were isolated according to the gating strategy as described in Supplementary Methods. A sample of the gating strategy is exemplified in Supplementary Fig. S4. The overall *in vitro* and *in vivo* work flow is explained in Supplementary Fig. S5.

### Bright phenotype of CD44 and EpCAM select for rapid growing NPC cells resulting in the formation of larger xenografts

**Figure 2:**
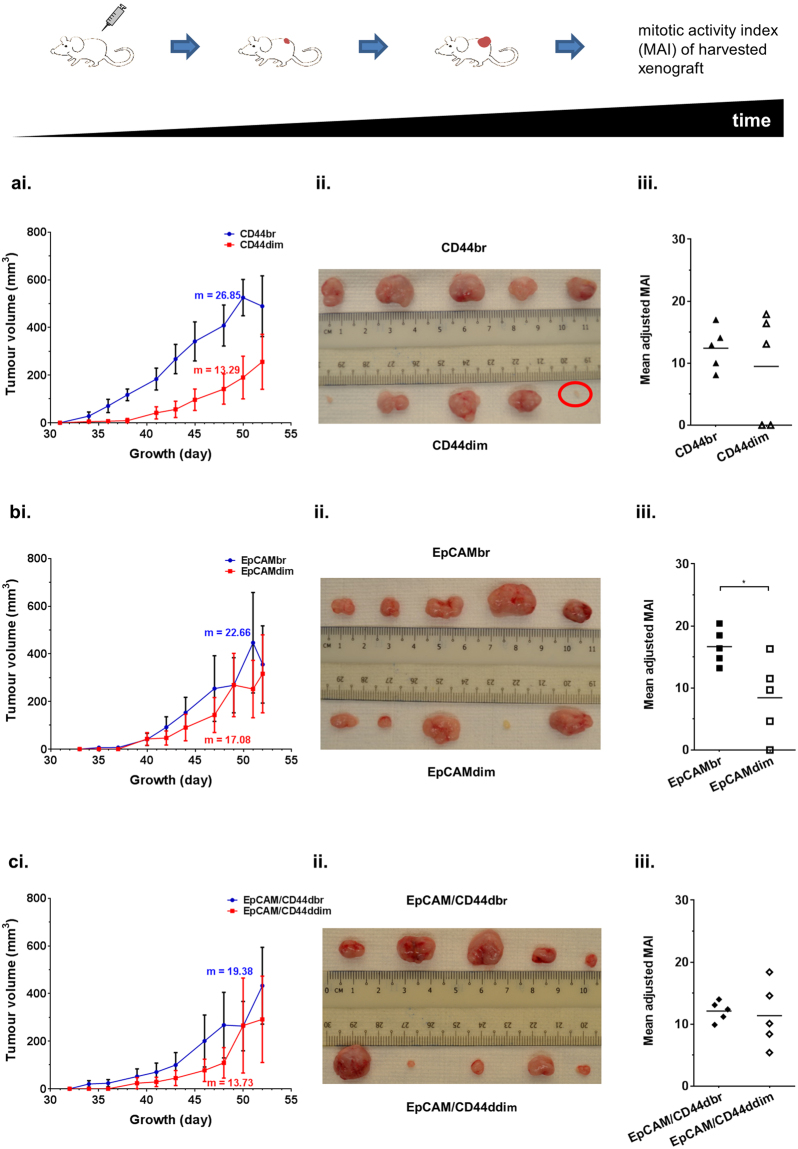
Growth properties of CD44, EpCAM and EpCAM/CD44 cells from C666-1. Growth curve (i), image (ii) and adjusted MAI (iii) of the harvested xenografts for (**a**) CD44, (**b**) EpCAM and (**c**) EpCAM/CD44. Results of growth curve, mean ± SEM of 5 tumour replicates. m, rate of volume increase. Red circle in (**a**ii) indicates a fat tissue and is considered as “no tumour”. *p < 0.05

Owing to an extremely low level of CD24 positive cells in C666-1 (Fig. 1), only CD44, EpCAM and EpCAM/CD44-selected cells from C666-1 were evaluated for their tumour-initiating ability in NSG mice. At an inoculation of 2,000 cells, all marker-selected C666-1 cells initiated 100% tumour formation (5/5) except for CD44dim (80%, 4/5) (Supplementary Table S2). CD44br cells significantly induced faster growth with a mean latency of 35.60 ± 1.50 days in contrast to CD44dim cells with a longer mean latency of 44.80 ± 6.85 days (p < 0.05) (Supplementary Table S2). Growth curve of CD44br-induced tumours was indicative of higher proliferation rate compared to the growth curve of CD44dim-induced tumours (Fig. 2ai). The mean adjusted mitotic activity index (MAI) for CD44br tumours was 124.20 ± 15.58 compared to 94.80 ± 39.47 for CD44dim tumours (Fig. 2aiii). Differences in mean latency data and growth curves were less apparent between EpCAMbr and EpCAMdim tumours (Supplementary Table S2, Fig. 2bi) but the significantly lower mean adjusted MAI in EpCAMdim tumours (p = 0.0299) was still observed (Fig. 2biii).

**Figure 3:**
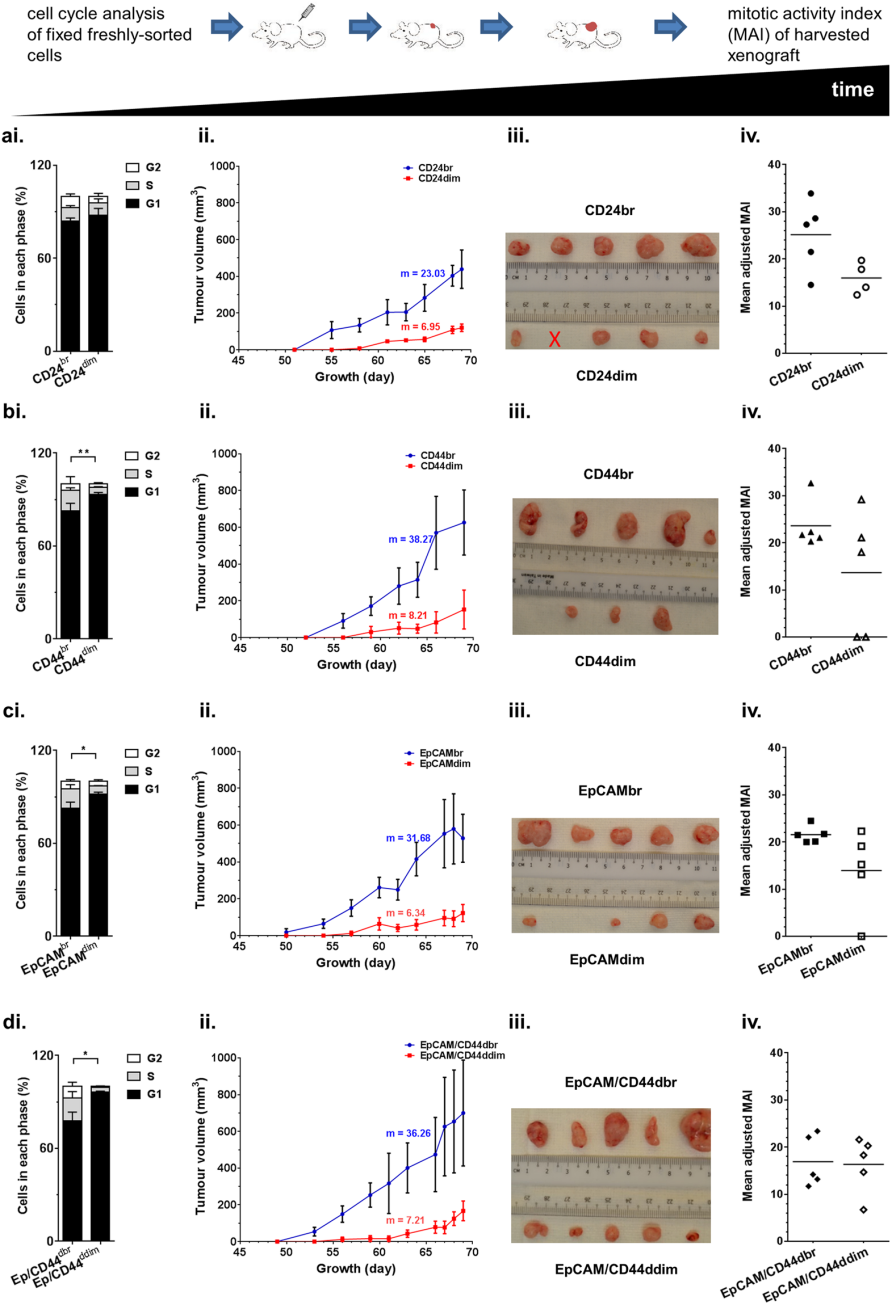
Growth properties of CD24, CD44, EpCAM and EpCAM/CD44 cells from xeno-B110. Cell cycle profile of freshly-sorted fixed cells (i), growth curve (ii), image (iii) and adjusted MAI (iv) of the harvested xenografts for (**a**) CD24, (**b**) CD44, (**c**) EpCAM and (**d**) EpCAM/CD44. Results of cell cycle profile, mean ± SD of 3 or 4 flow cytometry experiment replicates. *p < 0.05 (S-phase), **p < 0.01 (S-phase). Results of growth curve, mean ± SEM of 4 or 5 tumour replicates. m, rate of volume increase. X, mouse died immediately after inoculation.

As xeno-B110 is a NPC PDX newly established in our lab, a pilot experiment was performed to determine its tumour-forming ability with a titration of cell inoculation numbers (Supplementary Table S3). Host mouse cells (H2Kd positive) was removed by cell sorting and only viable non-mouse cells (H2Kd negative or “parental xeno-B110 cells”) were inoculated. There was a near 100% tumour formation from 100,000 to 500 cell inoculations (except for 5,000 cell inoculation). Tumour formation was greatly reduced at 100 cell inoculation (2/6; 33.33%) with no tumour at 10 cells (0/6; 0%). At 2,000 cell inoculation with marker-sorted cells, there was 100% tumour initiation (5/5 or 4/4) in all groups of cells except for CD44dim (60%, 3/5) and EpCAMdim (80%, 4/5) (Supplementary Table S4). Cell cycle analysis on fixed freshly-sorted cells showed that the percentage of S-phase cells were not significantly different between CD24br cells and CD24dim cells (Fig. 3ai). However, a 4-day shorter mean latency and slightly higher growth rate was observed in CD24br compared to CD24dim xenografts (Supplementary Table S4 and Fig. 3aii) which corresponded with a visible difference in mean adjusted MAI values within CD24 xenografts (Fig. 3aiv). CD44br cells contained 13.26 ± 1.56% of S-phase cells which were significantly higher compared to CD44dim cells which only had 4.41 ± 0.47% of S-phase cells (p < 0.01) (Fig. 3bi). The significant difference of mean latency between CD44br and CD44dim xenografts was 6.4 days (Supplementary Table S4). CD44br xenografts were larger and contained higher adjusted MAI than CD44dim xenografts (Fig. 3biii–iv). Similar to CD44br cells, EpCAMbr cells had significantly higher presence of S-phase cells (12.43 ± 2.77%) compared to EpCAMdim cells (5.06 ± 0.33%), a significant 8.6 days shorter mean latency, faster growth rate and higher adjusted MAI than EpCAMdim cells/xenografts (Supplementary Table S4, Fig. 3ci–iv).

Overall in both C666-1 and xeno-B110 cells, CD44br and EpCAMbr markers seemed to be consistently enriching for faster-growing cells. In order to investigate whether combination of CD44 and EpCAM markers may further enrich for faster-growing cells, C666-1 and xeno-B110 cells with double bright phenotype of EpCAM and CD44 (EpCAM/CD44dbr) were evaluated for their growth properties. Mean latency, growth curve and adjusted MAI’s differences were less discerning between EpCAM/CD44dbr and EpCAM/CD44ddim tumours from C666-1 (Supplementary Table S2, Fig. 2c). In xeno-B110, EpCAM/CD44dbr cells had 14.77 ± 4.15% of S-phase cells compared to EpCAM/CD44ddim cells which only had 3.22 ± 0.47% (p = 0.0425) (Fig. 3di). A significant 8.8 days difference in mean latency data was observed between EpCAM/CD44dbr cells and EpCAM/CD44ddim cells (Supplementary Table S4). A very obvious difference between the growth curves of EpCAM/CD44dbr and EpCAM/CD44ddim xenografts and the sizes of harvested xenografts were observed (Fig. 3dii–iii). However, there was no difference in the mean adjusted MAI between EpCAM/CD44dbr (169.20 ± 24.22) and EpCAM/CD44ddim (163.60 ± 26.51) xenografts (p = 0.8799) (Fig. 3div). Taken together, our data indicated that EpCAM/CD44dbr combination marker did not identify for a substantial increase of faster-growing cells than CD44br or EpCAMbr marker alone in C666-1 and xeno-B110.

### Significant enrichment of tumour-initiating cells (TICs) by CD44 and EpCAM in the first generation of xeno-B110

CD24br cells had a slight increase (1.79 folds) of TICs compared to CD24dim cells but it was not statistically significant (p = 0.42) (Table 1). CD44br and EpCAMbr cells were significantly more enriched in TICs than their respective dim phenotypes (17.49 folds at p < 0.001 and 4.97 folds at p = 0.01, respectively) (Table 1). The significant enrichment in TIC frequency was also observed in EpCAM/CD44dbr cells (8.25 folds at p < 0.01). However, it is noted that the enrichment fold of double bright markers did not exceed the one by single CD44br marker alone (Table 1).

**Table 1:** Limiting dilution assay for CD24, CD44, EpCAM and EpCAM/CD44 cells from xeno-B110 (first generation).

	Cell inoculation number	Number of tumours/Number of inoculated mice								
		H2Kd neg	CD24 br	CD24 dim	CD44 br	CD44 dim	EpCAM br	EpCAM dim	EpCAM/CD44dbr	EpCAM/CD44ddim
xeno-B110	30,000	4/4	3/3	3/3	3/3	3/3	3/3	3/3	3/3	3/3
	10,000	4/4	6/6	6/6	5/5	5/5	5/5	5/5	6/6	6/6
	5,000	5/6	5/5	6/6	4/4	4/4	6/6	6/6	5/5	5/5
	2,000	ND	5/5	4/4	5/5	3/5	5/5	4/5	5/5	5/5
	500	3/3	3/3	3/3	3/3	3/3	3/3	2/3	3/3	2/3
	100	2/6	2/6	1/6	5/6	1/6	3/6	2/6	5/6	1/6
	10	0/6	1/6	0/6	2/6	2/6	0/6	0/6	1/6	0/6
Estimated TIC frequency (95% CI)		1 in 1177 (393–3526)	1 in 159 (63–404)	1 in 285 (108–754)	1 in 45 (18–110)	1 in 787 (333–1859)	1 in 147 (58–373)	1 in 730 (308–1728)	1 in 56 (23–135)	1 in 462 (181–1181)
p value			0.42		<0.001		0.01		<0.01	
Enrichment factor (br/dim)			1.79		17.49		4.97		8.25	

ND, not determined.

### CD24, CD44 and EpCAM marker-sorted xeno-B110 cells retain self-renewal property during serial transplantation *in vivo*

An *in vivo* serial transplantation assay was performed using CD24, CD44 and EpCAM-selected cells from xeno-B110 to determine if they were able to self-renew by initiating new tumours up to the fourth generation. All groups were re-sorted for respective phenotype of cells before re-inoculation into recipient mice as secondary/tertiary/quartenary xenografts. All phenotypes could self-renew for at least four generations although with different TIC frequencies (Table 2) while maintaining similar histology of non-keratinizing differentiated NPC as the parental xeno-B110 cells (Supplementary Fig. S6). Upon serial transplantation at the fourth generation, CD24br cells appeared to have the highest enrichment of TICs (10.55 folds), followed by CD44br and EpCAMbr cells (7.07 and 4.89 folds, respectively) (Table 2).

**Table 2:** TIC frequency of CD24, CD44 and EpCAM cells from xeno-B110 at fourth generation.

	Cell Inoculation number	Number of tumours/Number of inoculated mice					
		CD24		CD44		EpCAM	
		br	dim	br	dim	br	dim
xeno-B110	500	3/3	1/3	3/3	2/3	3/3	2/3
	100	5/6	2/6	4/6	1/5	4/6	1/6
	10	1/6	0/6	2/6	0/6	0/6	0/6
Estimated TIC frequency (CI)		1 in 56 (23–135)	1 in 591 (177–1971)	1 in 67 (27–163)	1 in 474 (149–1511)	1 in 104 (42–261)	1 in 509 (162–1605)
p value		<0.01		<0.01		0.03	
Enrichment factor (br/dim)		10.55		7.07		4.89	

### CD24, CD44, and EpCAM-sorted xeno-B110 cells display differentially expressed genes and proteins

**Figure 4:**
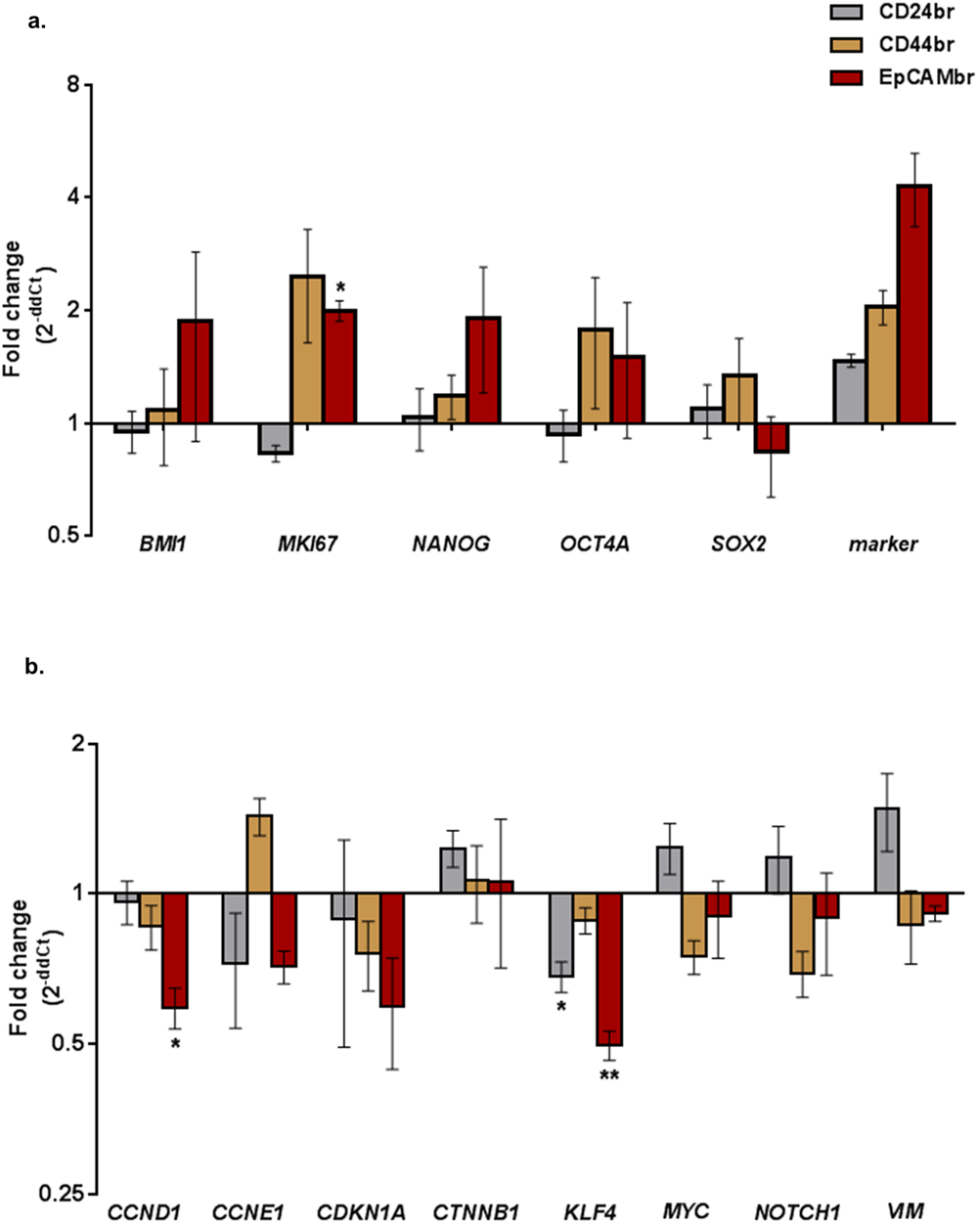
Gene expression of CD24br, CD44br, EpCAMbr and EpCAM/CD44dbr cells from xeno-B110. (**a**) Fold changes of *BMI1*, *MKI67*, *NANOG*, *OCT4A*, *SOX2*, *CD24*, *CD44* and *EPCAM* genes by RT-qPCR in ABI7500 FAST system. (**b**) Fold changes of *CCND1*, *CCNE1*, *CDKN1A*, *CTNNB1*, *KLF4*, *MYC*, *NOTCH1* and *VIM* genes by RT-qPCR in Fluidigm Biomark system. Results, (**a**) mean ± SEM of 3 sorted cell sample replicates, (**b**) mean ± SEM of 2 or 3 sorted cell sample replicates. *p < 0.05, **p < 0.01.

Expression of 21 genes was measured in freshly-sorted xeno-B110 cells: three housekeeping genes, three genes coding for surface markers used in this study, 15 genes associated with stemness, pluripotency, proliferation and cell cycle (Supplementary Table S5, Fig. 4). As expected, CD24br cells had higher *CD24* mRNA transcripts than CD24dim cells, *CD44* mRNA was more than 2-fold enriched in CD44br cells compared to CD44dim cells, whereas the levels of *EPCAM* mRNA transcripts in EpCAMbr cells were increased approximately 4 folds compared to EpCAMdim cells (Fig. 4a). Our data showed that overall the transcript levels of *NANOG* and *BMI* were higher in EpCAMbr cells compared to EpCAMdim cells (Fig. 4a). Compared to its counterpart, both EpCAMbr and CD44br cells had increased levels of *MKI67* and *OCT4A* (Fig. 4a). *KLF4* and its downstream transcriptional targets *CDKN1A* (encoding for p21) and *CCND1* (encoding for cyclin D1), *CCNE1*, and *VIM* were found to be differentially expressed in at least one of the marker sorted cells (Fig. 4b). *KLF4*, *CCND1* and *CDKN1A* transcripts were consistently downregulated in EpCAMbr cells compared to their respective dim phenotype. *CCNE1* level was slightly upregulated only in CD44br cells. Moderate upregulation of *VIM* was observed in CD24br cells. The levels of *CTNNB1*, *MYC* and *NOTCH1* were not changed in these sorted cells (Fig. 4b). *LMP1* and *LMP2A* mRNA transcripts from EBV were below the detection limit in this experiment (Ct > 35), although xeno-B110 was EBV positive as evident by positive staining for *EBER* (Supplementary Fig. S1). The scarcity of these two EBV transcripts was verified by RNA-ISH (data not shown).

**Figure 5:**
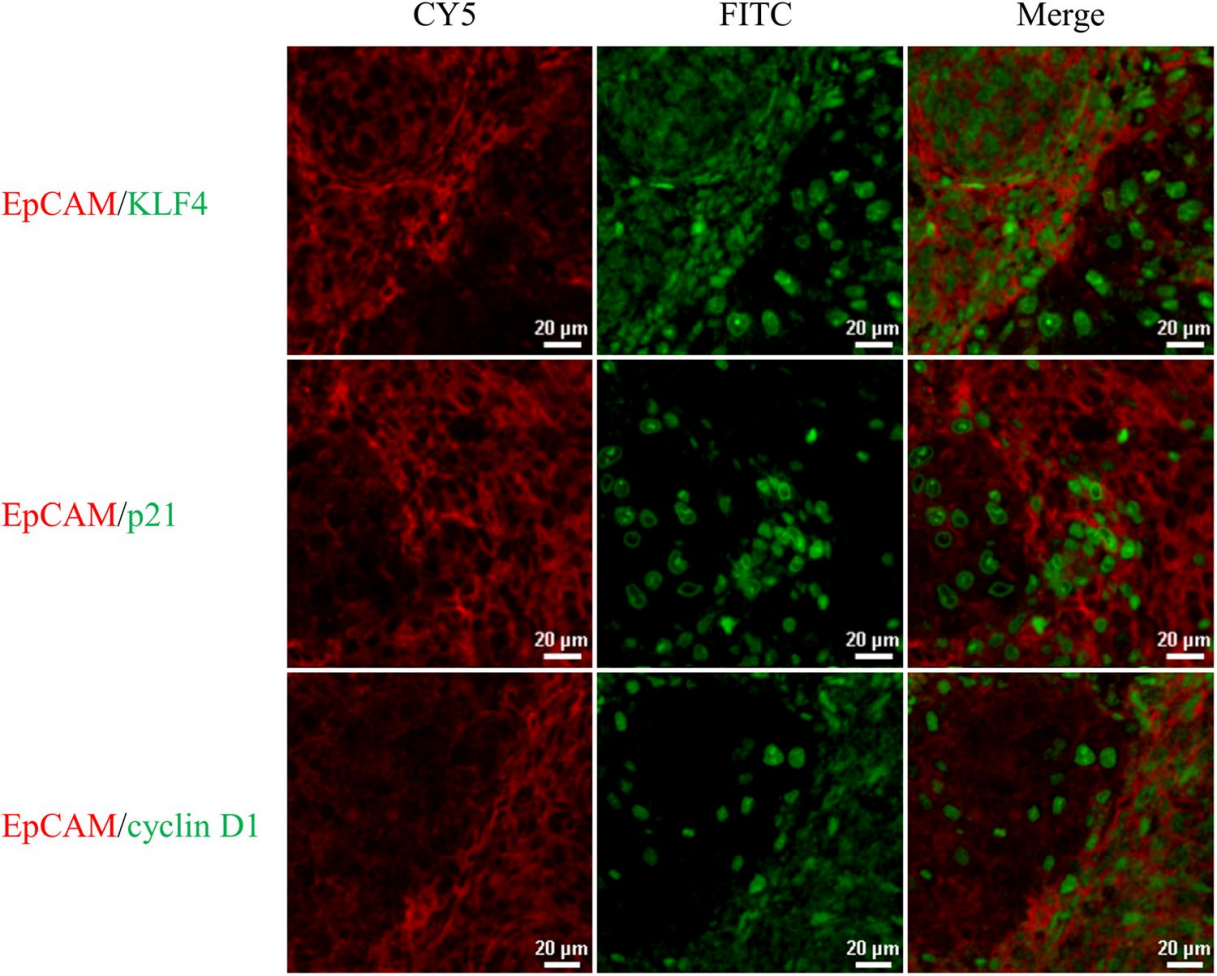
Co-expression of EpCAM with KLF4, p21 and cyclin D1 in xeno-B110. Representative images of EpCAM (CY5) co-stained with KLF4 (FITC), p21 (FITC) and cyclin D1 (FITC). 20X objective.

Downregulation of selected genes in subpopulations of marker-selected xeno-B110 cells was also seen at the protein level. The staining of freshly-sorted cytospin cells showed overall lower KLF4 in the bright cells compared to the corresponding dim cells (Supplementary Table S6). Co-staining of individual surface marker with KLF4 using immunofluorescence (IF) technique in xeno-B110 also revealed patches of marker bright area with low nuclear KLF4 staining, and vice-versa (representative images in Fig. 5, Supplementary Fig. S8). Although co-staining of surface markers with p21 or cyclin D1 in xeno-B110 tumour FFPE sections indicated inverse expression levels between these two proteins (representative images in Fig. 5), the differential p21 and cyclin D1 levels were less discerning in freshly-sorted cytospin cells (Supplementary Table S6).

### Modulation of KLF4 affects *in vitro* proliferation of xeno-B110

**Figure 6:**
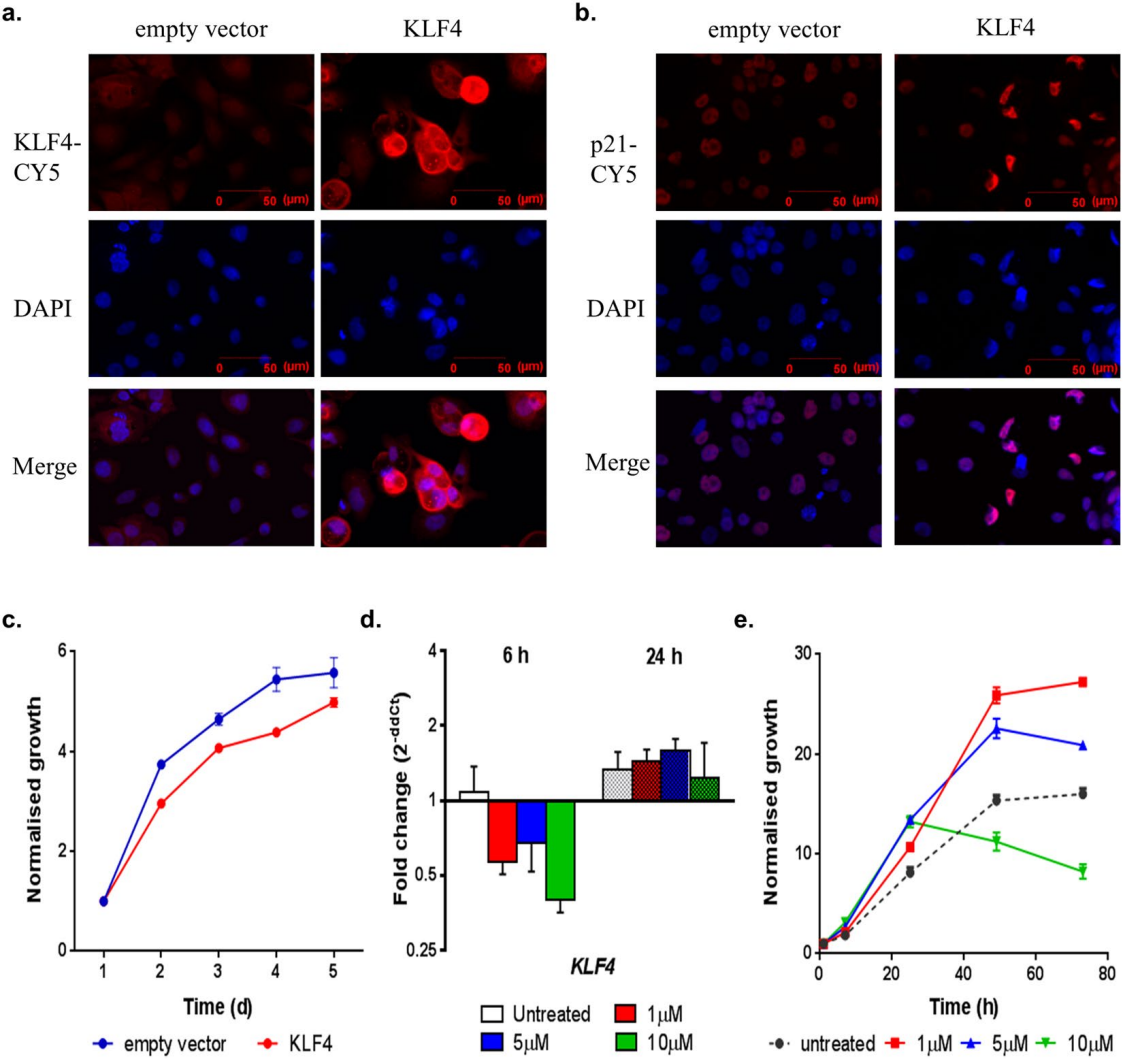
Modulation of KLF4 in xeno-B110. Overexpression of KLF4 was performed in EpCAMbr cells with KLF4 lentiviral construct and compared to control (empty vector). Representative images of transduced cells stained with (**a**) KLF4, (**b**) p21 and DAPI, 20X objective. (**c**) Representative *in vitro* growth curves of transduced EpCAMbr cells. (**d**) *KLF4* transcript levels at 6 and 24 hours after Kenpaullone treatment. (**e**) Representative *in vitro* growth curves of untreated and treated xeno- B110 cells. Results of growth curves, (**c**) mean ± SEM of 3 replicate wells, (**e**) mean ± SEM of 2 replicate wells. Results of gene expression fold change, (**d**) mean ± SEM of 3 sorted cell sample replicates.

EpCAMbr cells of xeno-B110 were transduced with KLF4 and evaluated for *in vitro* proliferation for 5 days. KLF4 overexpression was first verified in EpCAMbr cells transduced with KLF4 lentiviral construct compared to EpCAMbr cells containing empty vector (Fig. 6a). Functionally, there was a decrease in growth for KLF4-overexpressed EpCAMbr cells compared to EpCAMbr cells with empty vector (Fig. 6c). Consistent with this, there was an increased expression of p21, a downstream transcriptional target of *KLF4*, in KLF4-overexpressed EpCAMbr cells compared to EpCAMbr cells with empty vector (Fig. 6b).

Viable non-mouse xeno-B110 cells were treated with 0, 1, 5 and 10 μM Kenpaullone, a chemical inhibitor against KLF4 and evaluated for *in vitro* proliferation at 1, 6, 24, 48 and 72 h post-treatment. At 6 h post-treatment, *KLF4* mRNA was downregulated in Kenpaullone-treated cells in a dose-dependent manner compared to untreated cells (Fig. 6d). There was a corresponding increase of *in vitro* growth of these treated cells in comparison to untreated cells between 6 to 24 hours of treatment (Fig. 6e). At 24 h post-treatment, the transcripts level of *KLF4* seemed to have reverted to baseline which was comparable to the untreated cells (Fig. 6d). Lower dose-treated cells displayed an increase *in vitro* growth over that of untreated cells, whilst 10 μM treated cells had a decrease in cell growth (Fig. 6e). Overall, transient *KLF4* downregulation by Kenpaullone led to concurrent increase of *in vitro* growth.

### Expression of selected transcripts and proteins in NPC specimens

**Figure 7:**
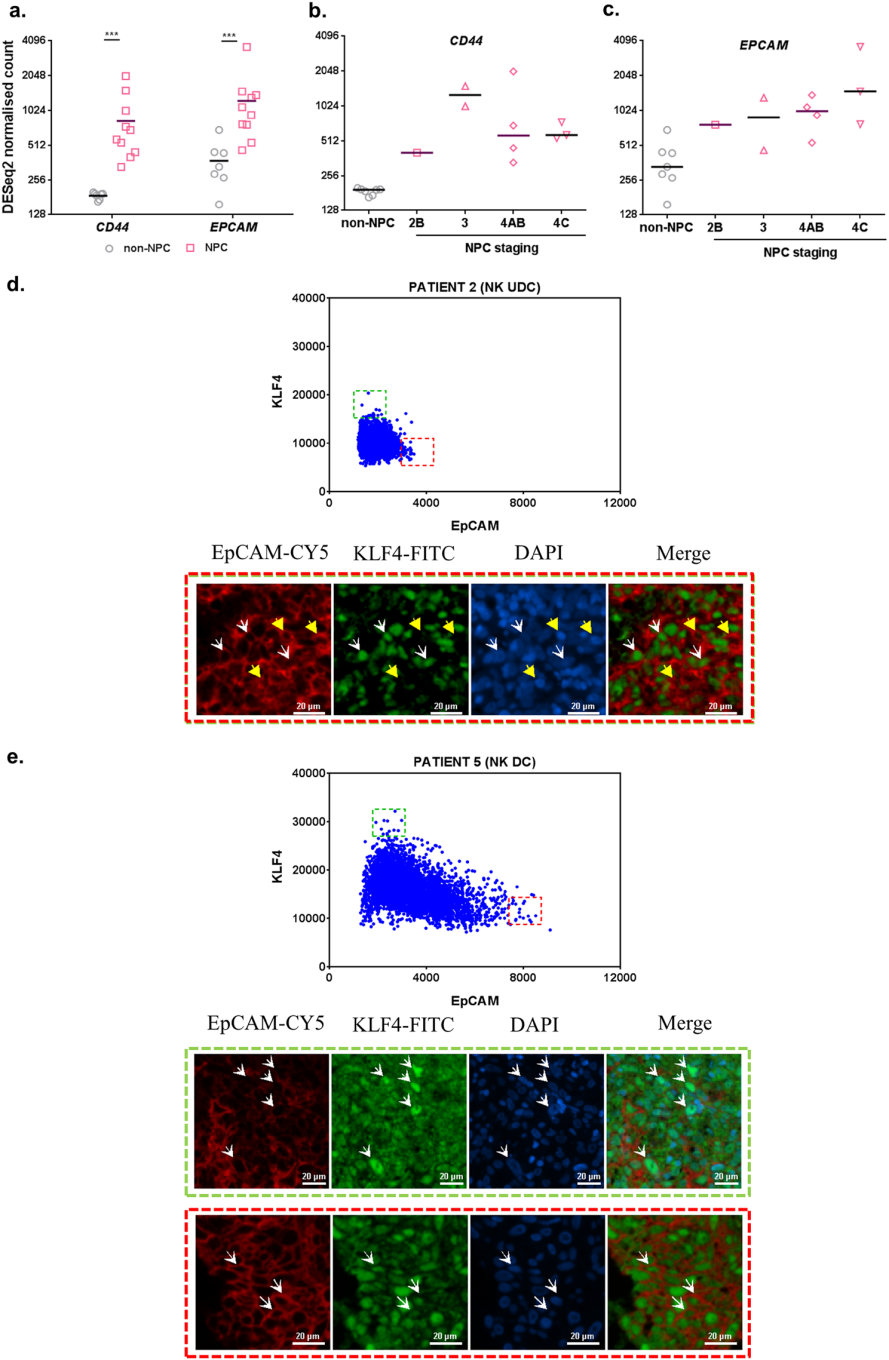
Expression of selected transcripts and proteins in non-NPC and NPC specimens. (**a**) RNA-seq data from non-NPC and NPC specimens were analysed for *CD44* and *EPCAM* expression. (**b**) *CD44* and (**c**) *EPCAM* expression was also individually analysed according to NPC disease staging. Results, median of 7 non-NPC or 10 NPC specimens for RNA-seq. (**d**) Co-staining of EpCAM and KLF4 in a patient specimen with non-keratinising (NK) undifferentiated carcinoma (UDC). Scatter plot of mean fluorescence intensities for EpCAM and KLF4 shows cells with high EpCAM and low KLF4 co-expressions highlighted in red dotted box, and low EpCAM high KLF4 cells highlighted in green dotted box. Yellow arrow head shows representative tumour cells from red dotted box. White arrow head shows representative tumour cells from green dotted box. (**e**) Co-staining of EpCAM and KLF4 in a patient specimen with non-keratinising (NK) differentiated carcinoma (DC). Scatter plot of mean fluorescence intensities for EpCAM and KLF4 shows cells with high EpCAM and low KLF4 co-expressions highlighted in red dotted box, and low EpCAM high KLF4 cells highlighted in green dotted box. Representative cells from red and green dotted boxes are highlighted by white arrow head in the corresponding images. All images were captured using 20X objective. ***p < 0.001.

Targeted RNA sequencing (RNA-seq) data was obtained from a prior study^36^ and reanalysed for specific transcripts using DESeq2 in 7 non-NPC and 10 NPC specimens (1 from non-keratinising differentiated carcinoma and 9 from non-keratinising undifferentiated carcinoma). *CD44* and *EPCAM* transcripts were significantly more than 2 folds upregulated in NPC compared to non-NPC specimens (p = 0.0001 for *CD44* and p = 0.0004 for *EPCAM*) (Fig. 7a). CD24 was not analysed due to failure of optimal primer design for CD24 in this targeted RNA-seq. CD44 expression was heterogeneously seen in NPC stages 2 to 4 (Fig. 7b). On the other hand, *EPCAM* expression increased with disease stage, with Stage 4 C having the highest expression (Fig. 7c).

Due to more promising tumourigenicity data, stable TIC frequency during serial transplantation and inverse association with *KLF4* mRNA level observed in EpCAMbr xeno-B110 cells, co-staining of EpCAM and KLF4 proteins were performed in a separate set of 10 archival specimens (3 from non-keratinising undifferentiated carcinoma, 6 from non-keratinising differentiated carcinoma and 1 from keratinising squamous cell carcinoma). Likewise in xeno-B110 tumour FFPE sections, the brightest stained tumour cells (above 95^th^ percentile) showed an inverse expression trend between the two proteins, i.e. high EpCAM and low KLF4 co-expressions, and vice-versa in the patient specimens (representative data in Fig. 7d-e).

## Discussion

This study attempts to delineate the biological properties of NPC cells identified by three common CSC surface markers concurrently within the same experiments. We first established that CD44br and EpCAMbr cells from both C666-1 cell line and xeno-B110 early-passage PDX consistently enriched for faster-growing tumourigenic cells which resulted in larger tumour growth, with more notable growth differences seen from xeno-B110 marker-selected cells. There was a higher TIC content in CD44br and EpCAMbr cells of xeno-B110 in the first generation of marker-induced growth; however, combination of CD44br and EpCAMbr markers did not further enhance for faster-growing cells or cells with higher TIC frequency than CD44br marker alone. CD24br, CD44br and EpCAMbr cells could self-renew for at least four generations. KLF4 was consistently downregulated in all bright phenotype of CD24, EpCAM and EpCAM/CD44 cells of xeno-B110 and was shown to be anti-proliferative in our *in vitro* study. Finally, heterogeneous KLF4 and EpCAM co-expression patterns were observed in archival NPC specimens and increased *EPCAM* coincided with increasing disease stage.

EpCAM has been in use as a CSC marker in other solid tumours such as breast, colon and pancreatic cancers since 2000s^37^. Clinically, high expression of EpCAM was also notably associated with higher gastric carcinoma cell proliferation and disease progression^38^. However, the role of EpCAM in NPC is still unclear. In our early-passage NPC PDX, xeno-B110, EpCAMbr marker identified for fast-growing cells with higher levels of stem cell related genes and had the ability to be passaged for at least four generations while maintaining its TIC frequency. Quantitative PCR and IF staining showed that EpCAMbr cells had lower *KLF4* (KLF4) compared to EpCAMdim cells in our NPC xenograft. Immunofluorescence staining in archival NPC specimens revealed that subsets of tumour cells co-expressing high EpCAM and low KLF4 protein levels were also present in NPC patients. Current knowledge suggests that advanced cancers contain higher number of CSCs than early cancers^39,40^. Indeed, our targeted RNA-seq analysis showed that *EpCAM* transcripts were increased with advanced stage of NPC, thus strengthening our view that EpCAM is a putative CSC marker in NPC.

CD44 is amongst the most investigated CSC markers in NPC^14^. This surface marker has been mainly studied in NPC cell lines such as C666-1, SUNE-1 and CNE-1^8,26,41^. The most striking differences between our study and currently available reports are i) our use of an early-passage NPC PDX as a study model, and ii) the evaluation of self-renewal ability which is central to (cancer) stem cell’s identity was assayed *in vivo* up to the fourth generation of serial transplantation. Our tumour initiation and *in vivo* growth data from CD44 xenografts of C666-1 and xeno-B110 are in agreement with prior studies. Many investigations on CD44 and self-renewal ability were performed in *in vitro* with spheroid or colony-forming assays^41–43^, whereas, we demonstrated that CD44br cells could self-renew *in vivo*. Our findings revealed that CD44br cells were proliferative in nature consistent with remarkably high S-phase cell content and increased *MKI67* mRNA transcript. There was a significant more than 2-fold increase of *CD44* transcript in NPC compared to non-NPC specimens in our study. However, we did not see a consistent trend of an increase of *CD44* in concurrence with lymph node involvement (data not shown) as reported in a prior study^44^.

CD24 as a CSC marker for NPC has received lesser attention than CD44. CD24^+^ was reported as a CSC phenotype in NPC cell lines TW02 and TW04^27^ while CD44^high^CD24^low^ nasopharyngeal epithelial cells transfected with LMP1 showed the ability to form tumour spheres *in vitro* ^45^. Nonetheless, CSC functionality i.e. *in viv*o self-renewal was not examined in both studies. Our findings revealed that CD24br cells showed slightly elevated level of *VIM*, a marker for cells undergoing EMT, and larger tumour growth that was not as marked as compared to other bright phenotypes. However, these CD24br cells could maintain tumour self-renewal ability *in vivo* for at least up to the fourth generation of serial transplantation and they warrant further investigations.

KLF4 is a transcription factor with known functions in pluripotency, tumour suppression or progression and cell differentiation^46^. There are a few lines of evidence in our study which imply that KLF4 has anti-proliferative effects in xeno-B110. CD24br, EpCAMbr and EpCAM/CD44dbr cells initiated larger xenograft growth, were more actively proliferating and had higher proportions of S-phase cells. These cells expressed decreased KLF4 (mRNA and protein) compared to their respective dim cells. The effect of KLF4 on NPC cell proliferation was validated by overexpression and inhibition studies. EpCAMbr cells of xeno-B110 which were transduced with KLF4 proliferated much slower than EpCAMbr cells transduced with empty vector. Conversely, transient inhibition of KLF4 with Kenpaullone (a short acting inhibitor of *KLF4* transcription^47^) in xeno-B110 cells indeed resulted in increased cell proliferation. The inverse relationship between *KLF4* levels and cell proliferation seen in xeno-B110 may be caused by the engagement of p53 by KLF4 to activate the transcription of *CDKN1A* gene which encodes p21, in turn leading to cell cycle arrest^46^.

CD24 positive and CD44 positive cells in HK1 was reported as 0.86% and 16.30%, respectively^27^. CD44 positivity in C666-1 ranged from 5% to approximately 45% in two independent studies^8,48^. We found that there was slightly more than 60% of CD24 positive and nearly 100% of CD44 positive cells in HK1, and more than 90% of CD44 positive cells in C666-1. The variations in immunophenotyping data between our study and others are believed to have arisen from technical differences such as culture conditions and enzymatic detachment, as well as gating strategy used to derive percentage of positive cells^49,50^.

In view of the limitation in long term cultured cell lines, early-passage PDX cells (passages 5 to 9) were used throughout our study to avoid losing the original identity and cellular features of the tumour^24,51^. The high tumourigenic ability (less cells needed to initiate tumour growth) seen in parental xeno-B110 cells may be explained by the probability of xeno-B110 itself being highly tumourigenic and the use of NSG mice in our study. Lacking functional natural killer (NK) cells in addition to mature T and B cells^52^, NSG mice provide highly efficient engraftment of exogenous cells as demonstrated by the seminal study of Quintana *et al*.^53^. The use of Matrigel as a co-inoculation agent in our study may have also improved tumour formation with as low as 100 cells. The ability of such low number of cells to form tumours has been reported. A 5-cell inoculation of melanoma cells mixed with Matrigel and injected into NSG mice had a tumour formation efficiency of 39% (7/18)^53^, whereas 100 cells of Matrigel-mixed CD44^+^ subpopulation and CD44^+^CD24^+^ESA^+^ subpopulation from pancreatic cancer PDXs formed tumours at an efficiency of 25% (4/16) and 50% (6/12), respectively^54^.

## Conclusion

In summary, CD44br and EpCAMbr cells from NPC cell line and early-passage PDX were fast-growing and more tumourigenic than their respective dim phenotype which resulted in larger tumours. However, the combination of CD44br and EpCAMbr markers did not further enrich for more fast-growing or tumourigenic cells. CD24br, CD44br and EpCAMbr cells from early-passage NPC PDX were also enriched for TIC and retained self-renewal property upon serial transplantation *in vivo*. The expression of *EPCAM* was negatively correlated with *KLF4* and *CDKN1A*, while KLF4 level was inversely associated with proliferation of NPC cells. Consistent with this, increased expression of *EPCAM* in NPC tumours was associated with more advanced stage of the disease. These suggest the importance of EpCAM in nasopharyngeal carcinoma.

## Materials and methods

### Ethics statements

All procedures and experimental protocols involving the use of NPC PDXs in this study were in accordance with the ethical standards of and approved by the Animal Care and Use Committee, Ministry of Health, Malaysia ((ACUC/KKM/02(3/2013) and ACUC/KKM/02(05/2016)). All procedures and experimental protocols involving the use of human specimens in this study were in accordance with the ethical standards of and approved by the Medical Research and Ethics Committee (MREC), Ministry of Health Malaysia (KKM/NIHSEC/P11-524 and KKM/NIHSEC/P13-108). Informed consent was obtained from all participating human subjects for the collection of fresh tissue and blood specimens. The use of anonymised archival tissue blocks was approved by the MREC.

### Cell lines and culture conditions

HK1 cells were cultured in RPMI-1640 medium containing 10% fetal calf serum and 1X penicillin/streptomycin (all from Gibco, USA) in 6-cm tissue culture plates (TPP, Switzerland). C666-1 cells were cultured in RPMI-1640 medium supplemented with 10% fetal calf serum, 1X Glutamax and 1X penicillin/streptomycin (all from Gibco, USA) in 10-cm tissue culture plates (BD Falcon, USA). Both cell lines were maintained in a 5% CO2 incubator at 37 °C. The cells were confirmed to be mycoplasma-free by periodical testing with Venor GeM Mycoplasma Detection Kit for Conventional PCR (Minerva Biolabs, Germany). Authentication of the cell lines (short tandem repeat, STR, profiling) were performed using the AmpFLSTR Identifiler PCR Amplification Kit (Applied Biosystem, Life Technologies, USA).

### Sample processing and staining for flow analyses or cell sorting

Freshly harvested xenografts (xeno-284 and xeno-B110) were digested and single cell suspensions of NPC cell lines and xenografts were stained and analysed or sorted in BD FACSAria SORP (BD Biosciences, USA).

### *In vivo* tumourigenicity

Four to 6 weeks old female NOD-scid gamma (NSG) mice (NOD.Cg-Prkdc^scid^ Il2rg^tm1Wjl^/SzJ; The Jackson Laboratory, USA) were used for endpoint experiments to measure tumour latency, growth curve and for calculation of mitotic figures with marker-selected C666-1 and xeno-B110 cells. Serial transplantation was also performed with marker-selected xeno-B110 cells.

Detailed protocols (above and others) are available in Supplementary Methods.

### Statistical analysis

Unpaired t-test was applied for mean latency, paired t-test for cell cycle and RT-qPCR, and Mann Whitney U-test for adjusted MAI, presence of necrosis and/or stroma and RNA-seq data using GraphPad Prism (version 6.0; GraphPad Software, Inc., USA). Significance was defined at the p < 0.05, p < 0.01 or p < 0.001 level as indicated in each figure description. Error bars represent mean ± SD or SEM as indicated in each figure description. TIC frequency was analysed according to Extreme Limiting Dilution Analysis (ELDA)^55^.

### Data availability

The dataset that supports the findings of RNA sequencing are available from Ching-Ching Ng upon reasonable request.

## Electronic supplementary material

Supplementary Information
