# Supplementary material for: CD24, CD44 and EpCAM enrich for tumour-initiating cells in a newly established patient-derived xenograft of nasopharyngeal carcinoma
Source: Sci Rep. 2017 Sep 28;7:12372. doi: 10.1038/s41598-017-12045-8 (PMC5620042; doi:10.1038/s41598-017-12045-8)
Supplement: Supplementary file 1 — Supplementary Information [file 41598_2017_12045_MOESM1_ESM.pdf]

CD24, CD44 and EpCAM enrich for tumour-initiating cells in a newly established patient-derived xenograft of nasopharyngeal carcinoma

Susan Ling Ling Hoe, Lu Ping Tan, Norazlin Abdul Aziz, Kitson Liew, Sin-Yeang Teow, Fazlyn Reeny Abdul Razak, Yoon Ming Chin, Nurul Ashikin Mohamed Shahrehan, Tai Lin Chu, Noor Kaslina Mohd Kornain, Suat-Cheng Peh, Cheng Eng Koay, Kwok-Wai Lo, Munirah Ahmad, Ching-Ching Ng, Alan Soo-Beng Khoo

Supplementary Methods

NPC patient-derived xenografts (PDXs)

Mouse husbandry

Four to 6 weeks old female NOD-scid gamma (NSG) mice (NOD.Cg-Prkdc^{scid} Il2rg^{tm1Wjl}/SzJ; The Jackson Laboratory, ME, USA) were used and housed in individually vented cages GM500 DGM (Tecniplast, PA, USA) with Pure-o'Cel bedding and Enrich-n'Nest paper chips (The Andersons Inc., OH, US) in the SPF Animal Facility with standard environmental conditions of temperature at 20 to 24°C, a relative humidity of 45 to 65% and a 12-h dark-light cycle. They had *ad libitum* access to sterile pelleted mouse feed and sterile acidic water (pH2.8 to 3.1) supplemented with co-trimoxazole (1:20 dilution).

A mouse would be sacrificed immediately if it showed signs of being unfit or moribund (even before the experiment endpoint was reached) such as big visible tumour (1,000-1,500 mm^3), significant weight loss (more than 15% body weight), any impairment of basic body functions (feeding, walking, etc.) and signs of continuous distress which could not be alleviated by other means.

Harvesting

Mouse bearing a tumour was checked for its correct identity via its ear tag. The mouse was humanely euthanized using CO_2 and/or cervical dislocation method. The exterior of the subcutaneous xenograft and its surrounding area was disinfected with 10% w/v Povidone iodine solution (Polylab, Malaysia). The xenograft was gently excised out and transferred to a sterile glass petri dish. Visible blood clots, blood capillaries and/or fat were removed. It was then cut length-wise to inspect for the presence of necrotic tissue which would be removed. A cross section of the xenograft was preserved in 10% neutral buffered formalin (Leica Biosystems, Newcastle, UK). The remaining xenograft was digested into single cell suspension for downstream experiments as previously described.

Digestion

Freshly harvested NPC PDX was rinsed several times with sterile cold PBS buffer supplemented with 1X antibiotic/antimycotic (Thermo Fisher Scientific Inc., MA, USA). It was minced into fine pieces in the presence of RPMI-1640 supplemented with 10% fetal calf serum and 1X penicillin/streptomycin (all from Thermo Fisher Scientific Inc., MA, USA) ("RPMI-10 medium") in a sterile 6-cm glass petri dish. The mixture was transferred to a 50-mL Falcon tube (BD Biosciences, MA, USA) and allowed to stand for 8 min at room temperature. Dead and dying cells present in the supernatant were removed and the remaining clumps of tissue pieces were transferred back to the glass petri dish. The tissue was digested in the presence of 1:1 ratio of RPMI-10 and collagenase/dispase (Roche Life Science, IN, USA) for xeno-284 and collagenase type II (Sigma-Aldrich, MO, USA) for xeno-B110 in the presence of 2U per mL of DNase I (Thermo Fisher Scientific Inc., MA, USA) to prevent clumping of undigested tissue pieces during digestion. Digestion was performed in 37°C 5% CO_2 incubator for 1 h on a medium speed rotating belly dancer. The slurry of cell suspension mixed with undigested pieces was filtered with a 40-µm cell strainer. The cell suspension was centrifuged at 900 rpm for 6 min at room temperature and the cell pellet was washed once in sterile PBS. The clean cell pellet was resuspended in 1 mL of RBC lysis buffer (Qiagen, Hilden, Germany) and incubated for 3 to 5 min at room temperature to lyse red blood cells. After centrifugation at 900 rpm for 6 min, the cell pellet was washed once in sterile PBS and spun again. The final cell pellet was resuspended in HBSS buffer (Thermo Fisher Scientific Inc., MA, USA) supplemented with 2% fetal bovine serum (Thermo Fisher Scientific Inc., MA, USA) and 10mM HEPES (Sigma-Aldrich, MO, USA) ("HBSS+ buffer") for cell and viability counts with trypan blue exclusion method.

Flow cytometry analysis or cell sorting

Staining of cells

C666-1 or HK1 cells in HBSS+ buffer were stained with CD24, CD44 or EpCAM antibody (Table 1) each at 10 μL per 10^6 cells, for 10 min at room temperature in the dark. Prior to antibody-staining, xenograft cell suspension in HBSS+ buffer was blocked with Fc receptor blocking reagents for mouse and human antibodies at a 1:10 ratio (Miltenyi Biotec, Bergisch Gladbach, Germany) for 10 min at room temperature. Fc receptor-blocked xenograft cell suspension was then stained with mouse H2Kd antibody and CD24, CD44, or EpCAM antibody each at 10 μL per 10^6 cells, for another 10 min at room temperature in the dark. Stained cells were washed once with sterile PBS and centrifuged at 900 rpm for 6 min at room temperature before being resuspended in HBSS+ buffer at a concentration of 1 X 10^6 cells per 500 μL for flow analysis or 5 to 8 X 10^6 cells per mL for cell sorting. The cells were then filtered with a 40-µm cell strainer before the addition of 0.2 µg/mL of DAPI as a viability dye as well as 10U per mL of DNase 1 to prevent cell clumping into the stained cell suspension. Unstained cells and cells stained with respective isotype control antibody (Table 1) were used as negative and gating controls, respectively.

<u>Flow cytometry data acquisition, cell sorting and post-sort analyses</u>

Data acquisition and cell sorting were performed on FACSAria SORP sorter equipped with 488-, 561-, 640- and 355-nm lasers (BD Biosciences, CA, USA). Fluorescence emission was measured with 530/30 (FITC), 582/15 (PE), 670/30 (APC) and 450/50 (DAPI) optical filters. Compensation was set up with CompBead Plus particles (BD Biosciences, CA, USA) singly-stained with the antibody being used. A small aliquot of cells (0.5 to 1 X 10^6 cells) heated at 95°C for 10 min were stained with DAPI and used to compensate for DAPI spillover. Cells stained with isotype-matched antibodies were used as gating controls.

Data acquisition, data analysis and cell sorting were performed using BD FACSDiva software (version 6.1.3; BD Biosciences, CA, USA). For the immunophenotyping of CD24, CD44 and EpCAM markers, a hierarchical gating strategy was employed to identify the

positive and negative marker expressions. Doublet discrimination gating was applied to remove doublets, followed by viable cell gating. Single viable cells were then gated for CD24, CD44 or EpCAM expression. The xenograft cells had an additional mouse cell exclusion gate (H2Kd negative) performed prior to gating for the individual surface marker. The expression levels for C666-1 and HK1 were reported as percentage of single viable cells, and for xeno-284 and xeno-B110 were given as percentage of single viable non-H2Kd cells. The experiment was repeated three times.

Hierarchical gating strategy was also employed in the cell sorting experiment to identify the bright and dim phenotypes of CD24, CD44, EpCAM and, combination of EpCAM and CD44 ("EpCAM/CD44") cells. Similarly, doublet discrimination gating was first applied followed by viable cell gating. Single live cells were then gated for H2Kd negative CD24, CD44, EpCAM or EpCAM/CD44 positive cells. The bright and dim phenotypes of each marker were gated from the marker positive cells and sorted or collected for downstream experiments. The expression levels were reported as percentage of single viable non-H2Kd marker bright ("markerbr") or dim ("markerdim") cells. Only the top total 5% of brightly-stained cells or the bottom total 5% of dimly-stained cells were regarded as markerbr or markerdim cells, respectively. Collection of sorted cells was performed at a sample flow rate of 1,000 to 1,500 cells per second at 4°C. Sorted cells were collected into 1.5-mL or 15-mL polypropylene tube containing approximately 400 μL or 3 mL of RPMI-10 medium, respectively. Typical collection time ranged from 5 min to slightly more than an hour, depending on the number of sorted cells needed for a downstream experiment.

Sorted cells were also periodically checked for post-sort purity. After collection, they were re-analyzed by flow cytometry and found to be typically above 90% purity (% from single viable non-H2Kd cells; representative data in Supplementary Fig. S4d). Their morphology

was also assessed microscopically. The majority of sorted cells were single, round and healthy-looking.

***In vivo* tumourigenicity**

Preparation of sorted cells for subcutaneous inoculation

Sorted cells in 1.5-mL or 15-mL polypropylene tube were centrifuged at 1,200 rpm for 6 min at room temperature. Supernatant was carefully aspirated out and the cell pellet was homogeneously mixed with 100 μL cold RPMI-1640 medium (Thermo Fisher Scientific Inc., MA, USA) and 100 μL cold Matrigel basement membrane matrix (BD Biosciences, CA, USA). Cold RPMI-1640 medium was then aspirated into a pre-chilled 1-mL syringe until the "0" graduated line, followed by the cell-Matrigel mixture. Presence of air bubble was removed by gently tapping the syringe to get the air bubble to the top of the syringe prior to pushing the bubble out with the plunger. The syringe containing cell suspension-Matrigel mixture was then placed in a near upright position (needle facing down) in an ice bucket and immediately transported to the SPF Animal Facility for mouse inoculation.

An endpoint experiment to measure tumour latency, growth curve and mitotic figures was performed with CD24, CD44, EpCAM and EpCAM/CD44 cells from C666-1 and xeno-B110. Two thousand markerbr/double markerbr, or markerdim/double markerdim cells were inoculated into five mice per group (a total of 7 groups for C666-1 and 8 groups for B110). Tumour volume measurements were recorded every two to three days using the formulae of ($width^2$ X length)/2. All animals (with and without tumour burden) were terminated at day 52 (C666-1 animals) or day 69 (xeno-B110 animals) post-inoculation when the majority of xenografts were below 800 mm^3. The rate of xenograft volume increase (m) was calculated from the slope of the best-fit line of a plot of volume versus time generated by a simple linear regression (modified from Blankenberg *et al.*[1]).

Serial transplantation was performed for CD24, CD44 and EpCAM cells from xeno-B110 to assay for self-renewal ability of sorted cells. Markerbr xenografts from the first generation were digested, stained and re-sorted for markerbr cells according to the above mentioned xenograft processing and flow cytometry procedures. The cells were then re-inoculated into recipient mice in a limiting dilution manner. Markerdim xenografts were also re-sorted for markerdim cells prior to re-inoculation in a limiting dilution manner. This was performed for three successive passages of xenografts. In order to avoid underestimating the frequencies of tumour-initiating cells (TICs)[2], mice with no tumour burden were kept for 150 days of observation prior to humane termination.

Tumourigenicity was measured by tumour incidence (i.e. number of tumours/number of inoculated mice) and latency (i.e. time from inoculation to time of first tumour measurements). Mean tumour volume per group was calculated as total volume of all xenografts divided by total inoculated mice. In the event of no measurable growth, the length of experiment was taken as the period of latency for the calculation of mean latency per phenotype for the animal, i.e. 52 days for C666-1 experiment, 69 days for xeno-B110 experiment and 150 days for serial transplantation experiment. Frequencies of TICs were calculated using Extreme Limiting Dilution Analysis (ELDA) software by Walter and Eliza Hall Institute, Australia (http://bioinf.wehi.edu.au/software/elda/)[3].

Data for CD24, CD44, EpCAM and EpCAM/CD44 animals were from one to three independent flow cytometry sorting experiments. The number of mice used in each experiment is indicated in the figures/tables.

Histology, scoring of mitotic figures, EBER-ISH, and cytokeratin IHC staining

Xenograft tissues were formalin-fixed for 24 h, embedded in automated tissue processor Leica ASP300 S (Leica Biosystems, Melbourne, Australia) and sectioned at 3-μm thickness. Haematoxylin and eosin (H&E) staining was performed using an autostainer Leica XL (Leica

Biosystems, Melbourne, Australia) and evaluated by a histopathologist. Mitotic figures were counted in 10 high power fields (HPFs; 40X objective) per sample using Leica DM1000 LED light microscope (Leica Microsystems, Singapore). Many xenografts contained necrotic and/or stromal cells despite their small volumes (Supplementary Fig. S7). Due to the presence of such cells and insufficiency of 10 HPFs in certain sections, mitotic activity index (MAI)[4] was normalised/adjusted to 100% presence of viable tumour cells ("normalised/adjusted MAI"). Mean adjusted MAI per group was calculated as total adjusted MAI from all scored xenograft sections divided by total number of inoculated mice.

Xenograft tissues which had been processed into formalin-fixed paraffin-embedded tissue blocks were sectioned at 3-µm thickness and prepared for EBER-ISH and cytokeratin IHC staining. Pre-treatment steps and staining were performed on the BondMax™ fully automated IHC and ISH immunostainer (Leica Biosystems, Melbourne, Australia).

Prior to hybridization with specific RNA probes (EBER, RNA positive control and RNA negative control), tissue sections underwent pre-treatment steps which included baking, dewaxing, rehydration and proteinase K enzymatic retrieval with Bond Enzyme Pretreatment Kit (Leica Biosystems, Newcastle, UK). Subsequently, tissue sections were hybridized with EBER probe (Bond ready-to-use ISH EBER Probe, Leica Biosystems, Newcastle, UK), RNA positive control probe (Bond ready-to-use ISH RNA Positive Control Probe, Leica Biosystems, Newcastle, UK) or RNA negative control probe (Bond ready-to-use ISH RNA Negative Control Probe, Leica Biosystems, Newcastle, UK) for 2 h at 37°C, followed by post-hybridization washing steps at ambient temperature. After hybridization, tissue sections underwent further staining processes which included peroxidase blocking (Bond Polymer Refine Detection, Leica Biosystems, Newcastle, UK) for 5 min at ambient temperature and incubation with anti-FITC/HRP antibody (1:50 dilution, Dako, CA, USA) for 20 min at ambient temperature. Visualization of HRP activity was carried out by incubation with DAB

chromogen (Bond Polymer Refine Detection, Leica Biosystems, Newcastle, UK) for 8 min at ambient temperature. Finally, tissue sections were counterstained using haematoxylin for 5 min. All staining steps were followed by washing steps using washing buffer or deionized water.

Cytokeratin IHC staining was performed with Bond Polymer Refine Detection System (Leica Biosystems, Newcastle, United Kingdom). The sections underwent pre-treatment steps which included baking at 60°C, dewaxing using Bond Dewax Solution (Leica Biosystems, Newcastle, UK) and rehydration with absolute ethanol (Merck Millipore, MA, USA). Tissue sections were then treated with antigen retrieval solution (Bond ER2, pH9, Leica Biosystems, Newcastle, UK) for 20 min at 98°C. Next, the sections were incubated with peroxidase blocking solution for 8 min at ambient temperature and followed by incubation with Rodent Block M blocking solution (Biocare Medical, CA, USA) for 15 min at ambient temperature. Subsequently, the sections were incubated with mouse anti-human cytokeratin (Table 1) for 15 min and followed by a second incubation with Rodent Block M blocking solution for 15 min at ambient temperature. This followed with incubation with post primary rabbit anti-mouse IgG for 8 min and incubation with the polymer of anti-rabbit poly-HRP-IgG for 8 min at ambient temperature. Finally, the expression of cytokeratin was visualized using DAB chromogen (staining of 10 min at ambient temperature), and haematoxylin was used as a counterstain (5 min at ambient temperature). All staining steps were followed by washing steps using either washing buffer or deionized water. After the final staining step, tissue samples were dehydrated by absolute alcohol (1 min) and clearing in xylene (3 min).

Cell cycle analysis

Markerbr or markerdim xeno-B110 cells (CD24, CD44, EpCAM and EpCAM/CD44) were centrifuged at 1,200 rpm for 6 min at room temperature. Supernatant was discarded until approximately 100 μL remained in the tube. This volume was used to resuspend the cell

pellet and the cell suspension was transferred to a 1.5-mL tube for a repeat centrifugation step. Supernatant was carefully aspirated without disturbing the cell pellet. Three hundred microlitres of cold 50% FBS in PBS was used to thoroughly resuspend the pellet. By using dropwise dispensing method, 900 μL of cold 70% ethanol was slowly added to the suspension with gentle mixing. The fixed cell suspension was incubated at -20°C until analysis. On analysis day, the fixed cells were washed with cold PBS twice to remove the ethanol and precipitated protein. Centrifugation was performed at 1,200 rpm for 6 min. The pellet was resuspended in cold PBS and filtered with a 40-μm cell strainer before the addition of 1 μg per mL of DAPI. Cell cycle analysis was performed on the FACSAria SORP sorter and fluorescence emission for DAPI was measured with 450/50 optical filter with a 355-nm laser. Percentage of cells in each cell cycle phase was analysed using ModFit LT software version 6 (Verity Software House, Inc., ME, USA). The experiment was repeated at least three times.

Reverse transcription (RT) quantitative PCR (qPCR)

RNA extraction

Total RNA from markerbr or markerdim xeno-B110 cells (CD24, CD44, EpCAM and EpCAM/CD44) were extracted using miRNeasy Micro kit (Qiagen, Hilden, Germany) following the manufacturer's protocol. Briefly, freshly-sorted cell pellets were lysed with QIAzol lysis buffer, homogenized and stored at -80°C until all replicates were collected. RNA extraction and on-column DNase digestion were performed accordingly with elution in 14 μL of nuclease-free water. Quantitation of RNA was performed using NanoDrop 8000 Spectrophotometer (Thermo Fisher Scientific Inc., MA, USA). Samples with contamination at 230- and/or 270-nm were cleaned using RNeasy® Plus Micro kit (Qiagen, Hilden, Germany) and re-quantitated. Total RNA from C666-1, HK1, HONE1, B95.8, SW480, Namalwa and MDA231 were extracted using RNeasy® Plus Mini kit (Qiagen, Hilden,

Germany) as per manufacturer's protocol and equally pooled to generate RNA positive control. Starting from 500 ng/μL of total RNA, a four-fold serial dilution of the RNA positive control sample was carried out to generate 16 standard points to be used in quality assessment of assays for gene expression study. All RNA samples were stored at -80°C until used.

RT-qPCR and data analysis of marker-selected cells using ABI7500 Fast system

cDNA was prepared from cell lysates using Cells-to-cDNA II Kit (Ambion, MA, USA) according to manufacturer's protocol. TaqMan assays and Fast Advanced Master Mix were used for qPCR in ABI7500 Fast Real-Time PCR System (Applied Biosystems, CA, USA) according to manufacturer's protocol. qPCR for each cell lysate was carried out in duplicate wells to evaluate the expression of *BMI1, MKI67, NANOG, OCT4A, SOX2, CD44, CD24 and EpCAM*. Negative control was carried out in each qPCR plate. Gene expression was normalised to housekeeping gene, *UBC*. Fold change was calculated using the formula of 2^{-ddCt}. One of the dim marker sorted cell replicates was used as calibrator to calculate fold change.

RT-qPCR and data analysis of marker-selected cells using Fluidigm Biomark system

RT was performed using Fluidigm® Reverse Transcription Master Mix (Fluidigm, CA, USA) as per manufacturer's protocol with 2 μL of each standard point, 4 ng of total RNA each from CD24br and CD24dim-sorted cells, and 8 ng of total RNA each from CD44br, CD44dim, EpCAMbr, EpCAMdim, EpCAM/CD44dbr and EpCAM/CD44dim-sorted cells. Nuclease-free water was used as a RT negative control.

Preamplification of 1.25 μL of each cDNA samples for 14 cycles was performed using Fluidigm® PreAmp Master Mix (Fluidigm, CA, USA) and pooled 0.2X TaqMan® gene expression assays (Thermo Fisher Scientific Inc., MA, USA) (refer to Supplementary Table 1

for list of assays). Nuclease-free water was used as preamplification negative control. Preamplified cDNA products were diluted 1:5 in TE buffer prior to qPCR.

qPCR was performed using the 48.48 or FLEXsix Dynamic Array integrated fluidic circuits (IFC), TaqMan® Universal PCR Master Mix (2X) (Thermo Fisher Scientific Inc., MA, USA) and TaqMan® gene expression assay (Thermo Fisher Scientific Inc., MA, USA) in the BioMark™ System (Fluidigm, CA, USA) as per manufacturer's protocol. RT negative control, preamplification negative control and a no-template qPCR control were included in each IFC so that non-specific amplification (if any) can be ruled out from further analysis. Data from standard points were used to construct standard curves for the evaluation of linear amplification in each assay. Three sorting replicates each for CD24, CD44 and EpCAM, and two sorting replicates for EpCAM/CD44 were analysed in this study. All qPCR reactions were performed in triplicate wells. List of genes assessed and used in the study is in Supplementary Table 2.

Fluidigm Real-Time PCR Analysis software was used to generate raw Ct values. Wells with undetected amplification (Ct = 999) or higher than 25 were excluded from calculation of average Ct values. Microsoft Excel was used to calculate average Ct values from duplicate or triplicate wells. Assays were excluded from data analysis when one of the followings was identified: (a) non-linearity was observed in the standard curves of serially-diluted pooled RNA positive controls ($R^2 \leq 0.9$), (b) PCR efficiency outside the range of 90-110% or (c) undetected amplification in more than 35% of all samples. Geometric mean of three reference genes *UBC*, *HPRT1* and *RPL13A* was used for data normalization. Gene expression was calculated with the formulae $2^{(-dCt)}$ whereby $dCt = Ct_{assay} - Ct_{geo\ mean\ RGs}$. The normalised values of markerbr were divided by markerdim to calculate fold change.

Immunofluorescence staining on xeno-B110 and NPC specimens

Multiplex immunofluorescence (IF) staining was performed on FFPE xeno-B110 tissue samples using the Opal staining system (Perkin Elmer, MA, USA). The antibodies and fluorophores used are shown in Table 1. Each surface marker was co-stained with a protein of interest as follows: EPCAM/KLF4, EPCAM/p21, EPCAM/CCND1; CD44/KLF4, CD44/p21, CD44/CCND1; CD24/KLF4, CD24/p21, CD24/CCND1. Prior to IF staining, tissue sections were baked at 60°C (1 hr), dewaxed in 3 changes of xylene (10 min each), and rehydrated through decreasing concentration of ethanol (1x absolute ethanol, 5 min; 1x 95% ethanol, 5 min; 1x 75% ethanol, 2 min), and washed with PBS. Next, tissue sections underwent microwave antigen retrieval step using citrate buffer. After the antigen retrieval, tissue sections were blocked using antibody diluent (10 min, room temperature), and then applied with the first primary antibody (20 min, room temperature). A secondary antibody conjugated to horse radish peroxidase (HRP) was applied (10 min, room temperature). Following this, a TSA-conjugated fluorophore (Opal 650) was added onto the tissue sections (10 min, room temperature). Then, the tissue samples underwent another cycle of microwave antigen retrieval step to strip the first primary and secondary antibodies, leaving the TSA-conjugated fluorophore bounded to the antigen site. The steps of blocking with antibody diluent until microwave antigen retrieval stripping were repeated for the second primary antibody and using Opal 540 TSA-conjugated fluorophore. Finally, tissue sections were counterstained with DAPI and cover-slipped before viewing. IF images were captured using QuantEM 512SC camera on a Nikon Ti-E widefield fluorescence microscope with 20x 0.75 NA objective and optimal fluorescence filter combinations which include DAPI filter (excitation: 325 –375 nm; emission: 435 – 485 nm, dichroic mirror: 400LP), FITC filter (excitation: 450 – 490 nm; emission: 500 – 550 nm, dichroic mirror: 495LP), and Cy5 filter (excitation: 590 – 650 nm; emission: 665LP, dichroic mirror: 660LP). Three different

regions of interest (ROI) were acquired per sample using similar camera settings and exposure time.

We further examined the expression levels of EpCAM and KLF4 in 10 archival NPC specimens. The protocol for staining and image capturing were as mentioned above. The tissue blocks of specimens used were confirmed to contain tumour tissue by H&E staining. For quantitative image analysis, three ROI were selected per specimen. These ROI were selected based on the following criteria: (i) regions contained tumour cells, and (ii) regions showed presence of cells positive for EpCAM. Multi-channel (DAPI/FITC/CY5) immunofluorescence images were analysed using NIS-Element software (version 4.2; Nikon Instruments Inc., NY, USA) for quantification of cell populations.

To prepare for analysis, unstained tissue (negative control) was used to define the background level or tissue auto-fluorescence. Next, using the 'bright spot detection' under the binary function, cells were defined and segmented by using DAPI counterstain to identify the nucleus and cytoplasm. Diameter of the cell, contrast, object symmetry and intensity were adjusted to achieve better cell segmentation. Using the 'Having' binary operation, 'whole cell' was defined by combining 'cytoplasm' and 'nuclei' segmentation.

After cell segmentation, thresholding was done for each channel to distinguish the object of interest from background. The minimum values were set at the maximum intensity detected for each channel on the negative control sample. Using the 'HAVING' binary operation, EpCAM positive ('EpCAM+') cells were defined by combining 'whole cell' with the 'EpCAM' (thresholded Cy5 channel) segmentation. Cells were further classified as EpCAM positive and KLF4 positive ('EpCAM+/KLF4+ cells') by combining 'EpCAM+ cells' and 'KLF4' (thresholded FITC) segmentation. Finally, the resulting 'EpCAM+/KLF4+ cells' binary layer was used to count objects using the 'automated measurement' function. All 10 archival NPC specimens were positive for EpCAM. Object data from the automated

measurement result were exported to Microsoft Excel for further analysis. GraphPad Prism (version 6.0; GraphPad Software, Inc., USA) was used to draw scatter plot for each specimen.

Immunocytochemical staining on marker-selected xeno-B110 cells

Preparation of cytospin slides from marker-selected cells

Marker-selected xeno-B110 cells of bright and dim phenotypes of CD24, CD44, EpCAM and EpCAM/CD44 were centrifuged at 800 rpm for 5 min at room temperature after cell sorting. Supernatant was decanted and the cell pellet was resuspended and fixed with 10% neutral buffered formalin for overnight at 4°C. The fixed cells were then washed twice with PBS prior to cytospin process onto silane-coated glass slides at 400 rpm for 5 min. The cytospin slides were air-dried for 2 h prior to use or to storage at -80°C.

Staining and scoring of KLF4, p21 and cyclin D1

Fixed cytospun bright and dim phenotypes of CD24, CD44, EpCAM and EpCAM/CD44 xeno-B110 cells were equilibrated to room temperature prior to permeabilisation with 0.5% Triton-X for 15 min at room temperature. Immunocytochemical (ICC) staining was then performed as described for cytokeratin IHC with the following modification and primary antibodies: antigen retrieval was performed with Bond ER2 (pH9, Leica Biosystems, Newcastle, UK) for 10 min at 98°C, rabbit anti-human KLF4, rabbit anti-human p21 Waf1/Cip1 and rabbit anti-human cyclin D1. Two pathologists reviewed the slides independently. Each slide was scored for percentage of positivity and intensity of stained cells as follows: <10% (0), 10-25% (1), 26-50% (2), 51-75% (3), >75% (4); negative to weak (1), moderate (2), strong (3). The sum of positivity and intensity was used as a total staining score for each protein. An overall total score of each protein was calculated by averaging the total staining scores from the two pathologists. The staining experiment was performed in one replicate due to technical limitation.

KLF4 experiments:

A. KLF4 overexpression

Lentiviral production and transduction

KLF4 lentiviral ORF Expression Clone (EX-Z5703-Lv105), empty control vector (EX-NEG-Lv105) and Lenti-Pac™ HIV Expression Packaging Kit were purchased from Genecopoeia (Rockville, MD, USA). To generate lentiviruses expressing either KLF4 or empty vector, HEK-293T cells (ATCC® CRL-3216™; American Type Culture Collection, VA, USA) were transfected with the above plasmid vectors using the lentiviral packaging kit as per manufacturer's instruction. The culture medium containing lentiviral particles was collected 48 hours post-transfection, aliquoted and stored at – 80°C prior to use. EpCAMbr cells from xeno-B110 were plated overnight at a density of 2 X 10^5 cells into ViewPlate-96 Black 96-well plates (PerkinElmer, MA, USA) coated with fibronectin (1 µg/cm^2) (Sigma, MO, USA). Plated cells were then transduced with lentivirus expressing either KLF4 or empty vector in the presence of polybrene (Sigma, MO, USA) at 10 µg/mL. After 24 hours, the lentiviral-containing medium was removed and replaced with fresh medium. For cell proliferation assay, transduced cells were incubated with fresh medium containing 1X RealTime-Glo™ MT Cell Viability Assay (Promega, WI, USA) reagent upon removal of lentiviruses. KLF4 overexpression efficiency was verified using immunofluorescence microscopy.

In vitro cell proliferation assay

Cell proliferation was studied using RealTime-Glo™ MT Cell Viability Assay (Promega, MI, USA) reagent as per manufacturer's instruction. Luminescence signal was measured daily for 7 days by the Envision multiplate reader (PerkinElmer, MA, USA) using the ultrasensitive luminescence detection mode. The medium containing 1X RealTimeGlo was changed every two days throughout the proliferation assay. Growth at each time point was normalised to that of time point day 1.

IF microscopy

EpCAMbr cells from xeno-B110 were plated as described in the "Lentiviral production and transduction" section for 48 hours. Cells which were lentiviral-transduced with either KLF4 or empty control vectors were maintained in the same 96-well plates for 120 hours post-transduction. Unless specified, all steps were carried out at room temperature. Cells were first washed once with PBS, fixed using 10% neutral-buffered formalin for 15 minutes, followed by three rinses with PBS. Cells were then permeabilised using 0.3% Triton X-100 for 10 minutes, washed thrice with PBS and blocked with 1% BSA in PBS for an hour. Upon completion of the blocking step, cells were incubated overnight with primary antibodies against KLF4 and p21 (Table 1) at 4°C. After three times of PBS washing to remove unbound primary antibodies, cells were incubated with appropriate Alexa Fluor 594-labeled secondary antibodies for one hour. Following that, cells were again washed thrice with PBS and cell nuclei were counterstained with DAPI (Sigma, MO, USA) for 10 minutes. Image acquisition was performed using the In Cell Analyzer 2000 equipped with a Nikon 20X/0.45 objective (GE Healthcare, NJ, USA). Images in the DAPI channel were acquired using 390/18 nm excitation and 432.5/48 emission filters with a 0.15-sec exposure time. Images in the Cy3 channel on the other hand were acquired using 542/27 nm excitation and 597/45 emission filters with a 1-sec and a 0.8-sec exposure time for KLF4 and p21 imaging, respectively.

B. Chemical inhibition of KLF4 using Kenpaullone

A black 96-well plate (Perkin Elmer, MA, USA) was pre-coated with 1μg/cm^2 fibronectin (Sigma Aldrich, IL, USA) at 4°C overnight and washed with 1X sterile PBS before use. Xeno-B110 was harvested and enzymatically digested into single cell suspension as described above. Viable non-mouse cells (DAPI negative and H2Kd negative cells) were sorted using FACSAria SORP. A total of 2.5 X 10^4 sorted cells were seeded into each well

of the coated plate in 100 μL of complete medium and cultured for 48 h. The media were then removed and Kenpaullone (Sigma-Aldrich, MO, USA) was added at final concentrations of 1, 5 and 10 μM in the presence of complete medium. Untreated control cells were treated with 0.01% DMSO. Cell proliferation was measured at 1, 6, 24, 48 and 72 h using EnVision Bioilluminator (Perkin Elmer, MA, USA) with the addition of 1X RealTime-Glo (Promega, WI, USA) onto the treated and untreated cells. Growth at each time point was normalised to that of time point 1 h. Each treatment condition was carried out in 2 replicate wells and evaluated in 3 independent sorting experiments. Within the same set of experiments, the cells were treated as described above, and they were lysed according to the manufacturer's protocol to evaluate the effect of Kenpaullone on *KLF4* mRNA expression in xeno-B110 cells. The cell lysates of 6 and 24 h post-Kenpaullone treatment were collected using resuspension buffer/lysis enhancer solution (Invitrogen, CA, USA) for RT-qPCR.

<u>RT-qPCR for cell lysates from Kenpaullone treatment using QuantStudio 12K Flex Real-time PCR system</u>

cDNA was prepared from cell lysates using Cells-to-cDNA II Kit (Ambion, MA, USA) according to manufacturer's protocol. TaqMan assays and Fast Advanced Master Mix were used for qPCR in ABI7500 Fast Real-Time PCR System or QuantStudio 12K Flex Real-Time PCR System (Applied Biosystems, CA, USA) according to manufacturer's protocol. qPCR for each cell lysate was carried out in duplicate wells to evaluate the expression of *KLF4*. Negative control was carried out in each qPCR plate. Gene expression was normalised to housekeeping gene, *UBC*. Fold change was calculated using the formulae of $2^{(-ddCt)}$. One of the untreated cell replicates was used as calibrator to calculate fold change.

RNA sequencing

RNA-seq data from a previous study was used[5]. NPC and healthy nasopharynx tissue recruitment, processing and RNA isolation for gene expression analysis were previously

described[5]. Differential gene expression analysis was performed in DESeq2[6]. DESeq2 normalised read counts were plotted in GraphPad Prism (version 6.0; GraphPad Software, Inc., USA).

Table 1: List of antibodies used.

Experiment	Antibody	Dilution used	Host species	Clone	Brand
Flow analyses/cell sorting	CD24-PE	1:10	Mouse	ML5	BD Biosciences, CA, USA
	CD44-PE	1:10	Mouse	G44-26	BD Biosciences, CA, USA
	EpCAM-APC (CD326)	1:10	Mouse	HEA-125	Miltenyi Biotec, Germany
	H2Kd-FITC	1:10	Mouse	SF1-1.1	BD Biosciences, CA, USA
	IgG2a,κ-FITC/PE	1:10	Mouse	G155-178,	BD Biosciences, CA, USA
	IgG2b,κ-PE	1:10	Mouse	27-35	BD Biosciences, CA, USA
	IgG1-PE	1:10	Mouse	NA	Miltenyi Biotec, Germany
	IgG2b-FITC	1:10	Mouse	GC198	BD Biosciences, CA, USA
	IgG1-APC	1:10	Mouse	NA	Miltenyi Biotec, Germany
Immunohistochemistry/ immunocytochemistry	Cytokeratin	1:100	Mouse	AE1/AE3	Dako, CA, USA
	KLF4	1:200	Rabbit	H-180	Santa Cruz, TX, USA
	p21 Waf1/Cip1	1:200	Rabbit	12D1	Cell Signaling Technology, MA, USA
	Cyclin D1	1:100	Rabbit	92G2	Cell Signaling Technology, MA, USA
Immunofluorescence (Opal)	EpCAM	1:100	Rabbit	EPR677	Abcam
	CD44	1:100	Mouse	156-3C11	Cell Signaling
	CD24	1:100	Rabbit	NA	Abcam
	KLF4	1:100	Rabbit	NA	Santa Cruz

	p21	1:50	Rabbit	12D1	Cell Signaling
	Cyclin D1	1:50	Rabbit	92G2	Cell Signaling
Immunofluorescence (KLF4 overexpression)	EpCAM,	1:100	Mouse	Ber-EP4	Dako, CA, USA
	KLF 4	1:50	Rabbit	H-180	Santa Cruz Biotechnology, CA, USA
	p21 Waf1/Cip1	1:100	Rabbit	12D1	Cell Signaling Technology, MA, USA
	Mouse IgG (H+L) cross-adsorbed secondary antibody-Alexa Fluor 594	1:500	Goat	NA	Invitrogen, CA, USA
	Rabbit IgG (H+L) cross-adsorbed secondary antibody-Alexa Fluor 594	1:500	Goat	NA	Invitrogen, Carlsbad, CA, USA

NA, not available

Supplementary references

1. Blankenberg, F. G. *et al.* The influence of volumetric tumor doubling time, DNA ploidy, and histologic grade on the survival of patients with intracranial astrocytomas. *Am. J. Neuroradiol.* **16,** 1001–1017 (1995).
2. Quintana, E. *et al.* Efficient tumour formation by single human melanoma cells. *Nature* **456,** 593–598 (2008).
3. Hu, Y. & Smyth, G. K. ELDA: Extreme limiting dilution analysis for comparing depleted and enriched populations in stem cell and other assays. *J. Immunol. Methods*

347, 70–78 (2009).

4. Medri, L. Prognostic relevance of mitotic activity in patients with node-negative breast cancer. *Mod. Pathol.* **16,** 1067–1075 (2003).

5. Chin, Y.-M. *et al.* Integrated pathway analysis of nasopharyngeal carcinoma implicates the axonemal dynein complex in the Malaysian cohort. *Int. J. Cancer* **139,** 1731–1739 (2016).

6. Love, M. I., Huber, W. & Anders, S. Moderated estimation of fold change and dispersion for RNA-seq data with DESeq2. *Genome Biol* **15,** 550 (2014).

Supplementary Tables and Figures

Cell line / STRs	C666-1[1]	C666-1[2]	HK1[1]	HK1[2]	Patient's sample	xeno-284	Patient's sample	xeno-B110
**D5S818**	11, 12	11	11, 13	11, 13	11	11	12	12
**D13S317**	8, 11	8, 11	11	11	12	12	8, 9	8, 9
**D7S820**	11, 12	11, 12	8, 11	8, 11	10, 11	10, 11	10, 11	10, 11
**D16S539**	10	10	9, 11	9, 11	11, 12	11, 12	10	10
**vWA**	17, 18	17, 18	18, 19	18, 19	17, 19	17, 19	18, 20	18, 20
**THO1**	6, 8	6, 8	7, 9	7, 9	9, 10	9, 10	6.3, 8.3	9
**TPOX**	8, 11	8, 11	8, 11	8, 11	8	8	11	11
**CSF1PO**	11, 15, 16	11	11, 12	11, 12	10	10	11	11
**D3S1358**	16, 17	16, 17	15	15	16, 18	16	15, 16	16
**FGA**	23, 24	24	26	26	19	19	23, 24	19, 23, 24
**D8S1179**	11, 13, 14, 15	11, 15	11, 13	11, 13	14	14, 15	15	15
**D21S11**	28, 29, 30.2, 31.2	28, 31.2	28, 29	28, 29	29, 30	29	29, 33.2	29, 33.2
**D18S51**	16	16	17	17	14, 17	14, 17	14, 20	14, 20
**D19S433**	13, 15.2	13, 15.2	14	14	13	13	13	13
**D2S1338**	16, 23	15, 16, 23	20, 23	20, 23	19, 20	19, 20	17, 23	17, 23
**Amelogenin**	X,Y	X, Y	X	X	X, Y	X	X, Y	X, Y
**% Match**		85.7		100		91.7		90.2

[1] published data from Chan et al.[30]
[2] data from cell line used in our lab

Table S1 | Short tandem repeat (STR) profiling data of C666-1, HK1, xeno-284 and xeno-B110.

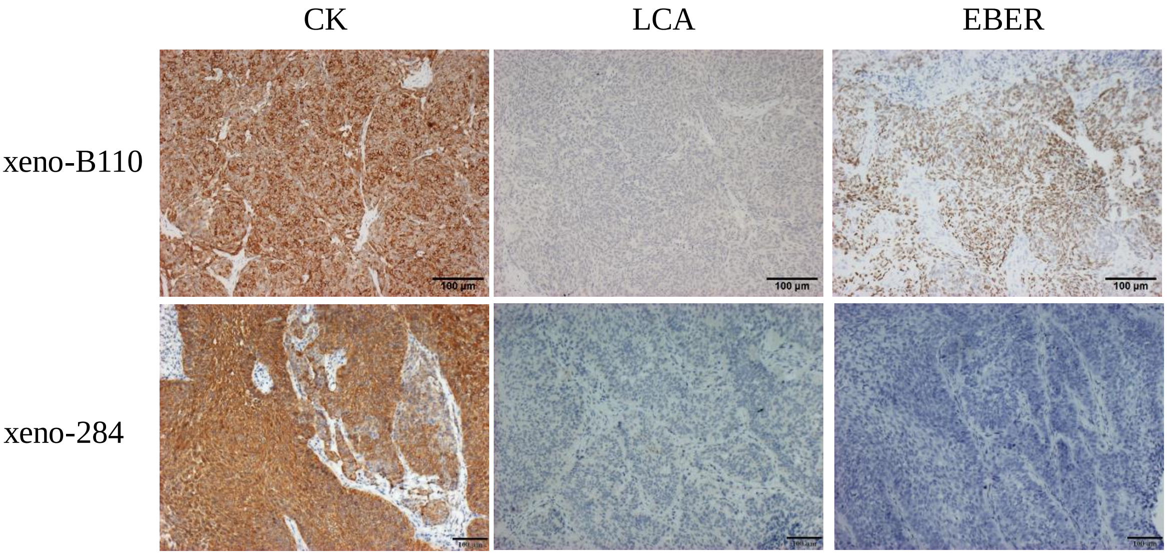

Figure S1 | Presence of cytokeratin, leucocyte and EBER in xeno-B110 and xeno-284. Representative images of xeno-B110 and xeno-284 tissue samples stained with cytokeratin (CK), leucocyte common antigen (LCA) and Epstein-Barr encoded RNA (EBER) antibody/probe. 20X objective; scale bar 100 µm.

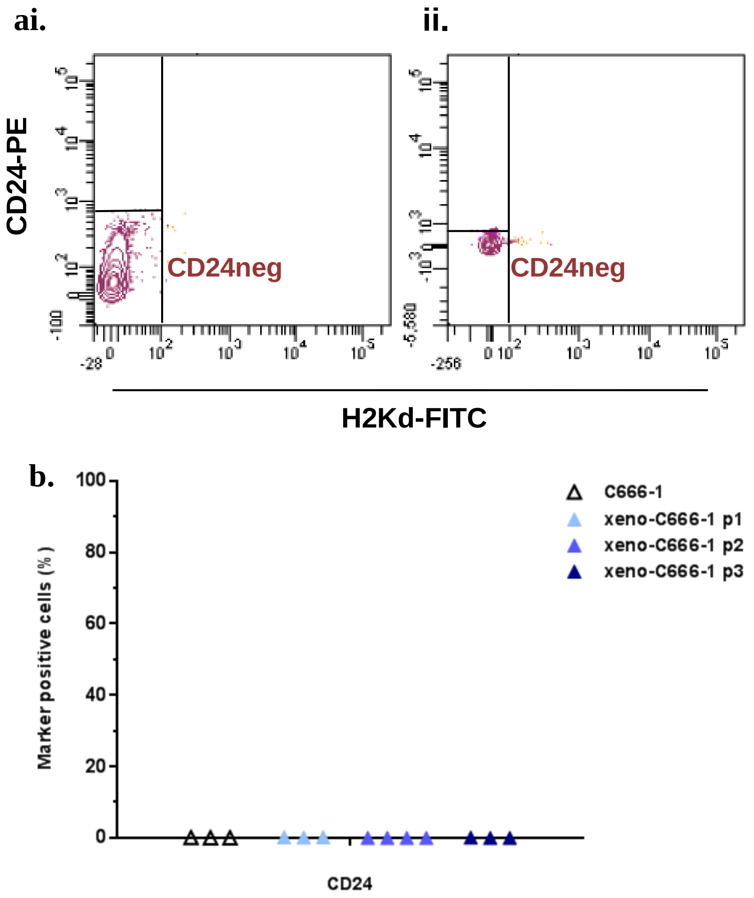

Figure S2 | Immunophenotyping of CD24 expression in C666-1 and xeno-C666-1. (**a**) Representative contour plots of immunophenotyping data for xeno-C666-1 cells stained with isotype control (i) and CD24 antibody (ii). (**b**) Percentage of marker positive cells from C666-1 was counted from the total number of single, viable cells. The denominator for xeno-C666-1 passages 1 to 3 was total number of single, viable, non-mouse cells.

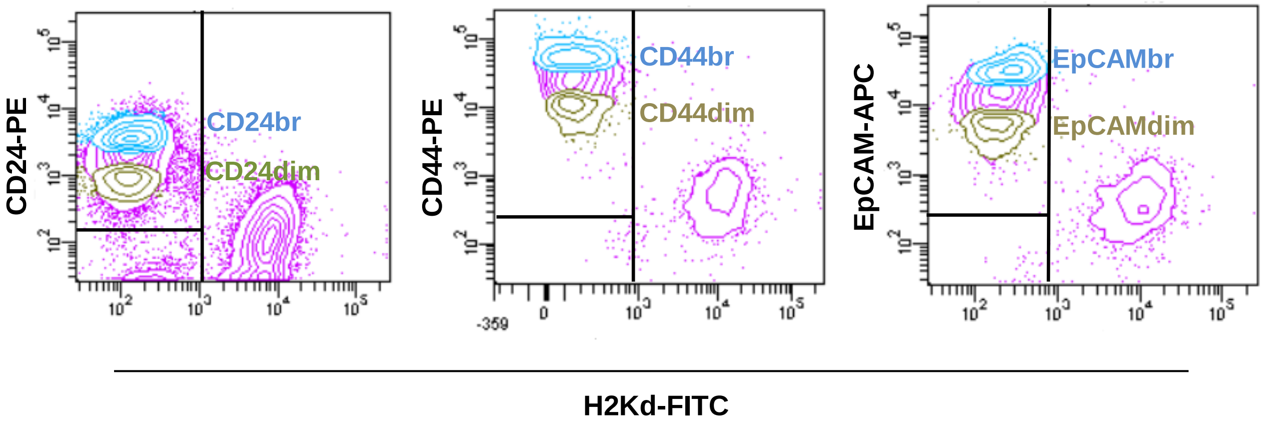

Figure S3 | xeno-B110 contains differential expressions of CD24, CD44 and EpCAM. Representative contour plots of immunophenotyping data for CD24, CD44 and EpCAM in xeno-B110 cells. Contaminating host mouse cells were identified by H2Kd antibody. Non-mouse CD24^{neg}, CD44^{neg} and EpCAM^{neg} cells were too few to be sorted.

a.

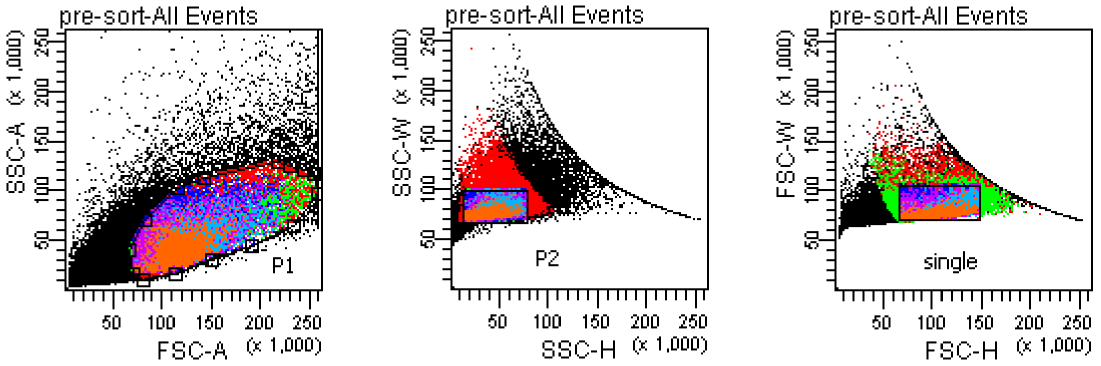

b.

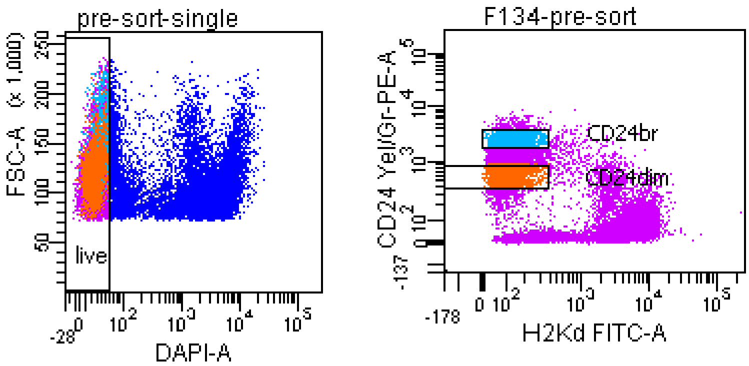

c.

Tube: pre-sort

Population	#Events	%Parent	%Total
All Events	154,764	####	100.0
P1	40,631	26.3	26.3
P2	36,928	90.9	23.9
single	31,742	86.0	20.5
live	20,000	63.0	12.9
CD24br	1,853	9.3	1.2
CD24dim	2,164	10.8	1.4

d.

**Marker**	**Post-sort purity, % (total number of cells analysed)**	
	bright/ double bright	dim/ double dim
CD24	91.8 (676)	92.0 (677)
CD44	94.0 (961)	94.4 (663)
EpCAM	91.5 (257)	92.8 (770)
EpCAM/CD44	90.8 (226)	90.7 (261)

Figure S4 | Hierarchical gating strategy employed in xeno-B110 sorting experiments. (**a**) Representative dot plots show how single cells were gated from freshly-digested and stained xeno-B110 cell suspension using the doublet discrimination gating strategy available in BD FACSDiva software. (**b**) Single cells were then gated for live (viable) cells with DAPI dye, followed by gating for H2Kd (mouse) negative markerbr (CD24br in this figure) and markerdim (CD24dim in this figure) cells for sorting. (**c**) Overall gating strategy and the respective number or percentage of cells (events) for each gate in the shown dot plots are presented. (**d**) Sorted cells were also periodically checked for their purity and were typically above 90% purity.

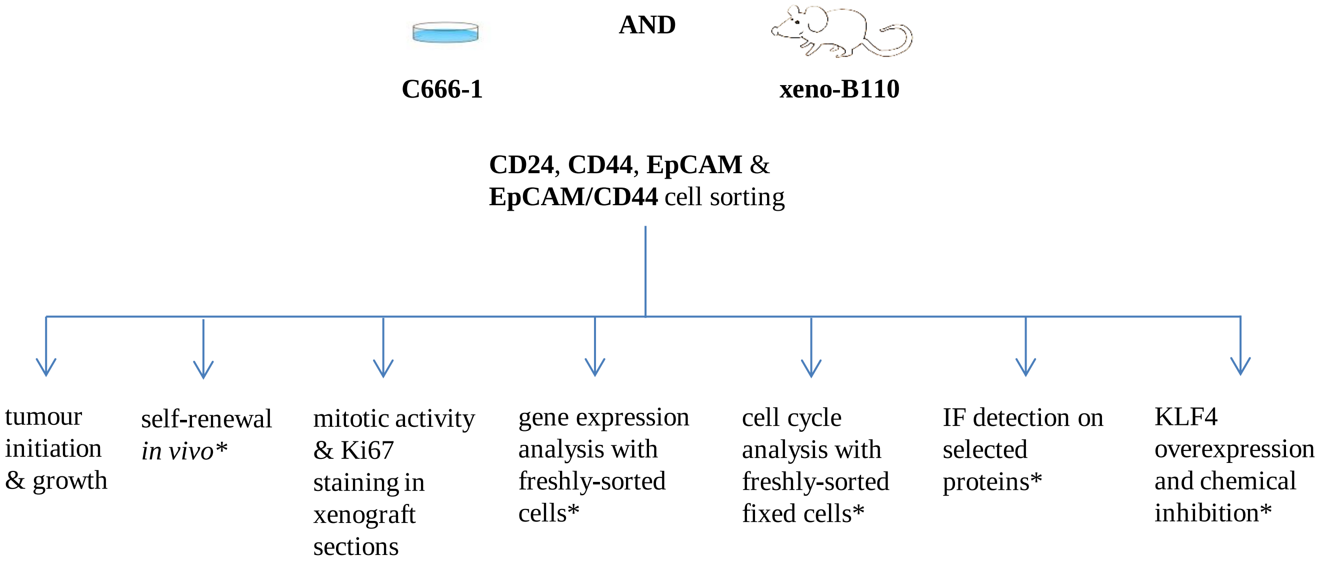

Figure S5 | Schematic work flow of *in vitro* and *in vivo* studies

* only xeno-B110 cells were evaluated

Sample	Phenotype	Number of tumours/Number of mice	Mean latency ± SD (days)	p value
		2,000 cells		
C666-1	viable	5/5	42.00 ± 5.55	
	CD44br	5/5	35.60 ± 1.50	0.03
	CD44dim	4/5#	44.80 ± 6.85	
	EpCAMbr	5/5	40.60 ± 3.07	0.14
	EpCAMdim	5/5	45.20 ± 4.62	
	EpCAM/CD44dbr	5/5	40.60 ± 5.85	0.21
	EpCAM/CD44ddim	5/5	46.00 ± 5.40	

mouse with no tumour was given latency of 52 days (duration from inoculation until termination of experiment)

Table S2 | Tumour initiation and mean latency data of xenografts arising from bright and dim phenotypes of CD44, EpCAM and EpCAM/CD44 cells of C666-1. Results, mean ± SD of 5 replicates per inoculation.

Sample/Phenotype	Number of cells inoculated	Number of tumours/Number of mice	Mean latency ± SD (days)
xeno-B110/H2Kdneg	100,000	4/4	63.50 ± 1.00
	50,000	4/4	71.75 ± 13.70
	30,000	4/4	71.00 ± 6.93
	10,000	4/4	78.75 ± 11.06
	5,000	5/6*	74.83 ± 37.74
	500	3/3	70.33 ± 14.57
	100	2/6*	126.00 ± 37.71
	10	0/6*	150.00 ± 0.00

* mouse with no tumour was given latency of 150 days (duration from inoculation until termination of experiment)

Table S3 | Tumour initiation and mean latency data of the xenografts arising from limiting dilutions of H2Kd negative cells of xeno-B110. Results, mean ± SD of 3-6 replicates per inoculation.

Sample	Phenotype	Number of tumours/Number of mice<br>2,000 cells	Mean latency ± SD (days)	p value
xeno-B110	CD24br	5/5	56.20 ± 2.68	0.03
	CD24dim	4/4^	60.25 ± 1.50	
	CD44br	5/5	57.80 ± 2.68	0.03
	CD44dim	3/5*	64.20 ± 4.55	
	EpCAMbr	5/5	54.40 ± 3.58	0.02
	EpCAMdim	4/5*	63.00 ± 5.61	
	EpCAM/CD44dbr	5/5	54.80 ± 2.68	<0.01
	EpCAM/CD44ddim	5/5	63.60 ± 4.93	

^ 1 mouse died after inoculation

* mouse with no tumour was given latency of 69 days (duration from inoculation until termination of experiment)

Table S4 | Tumour initiation and mean latency data of xenografts arising from H2Kd negative bright and dim phenotypes of CD24, CD44, EpCAM and EpCAM/CD44 cells from xeno-B110. Results, mean ± SD of 4 or 5 replicates per inoculation.

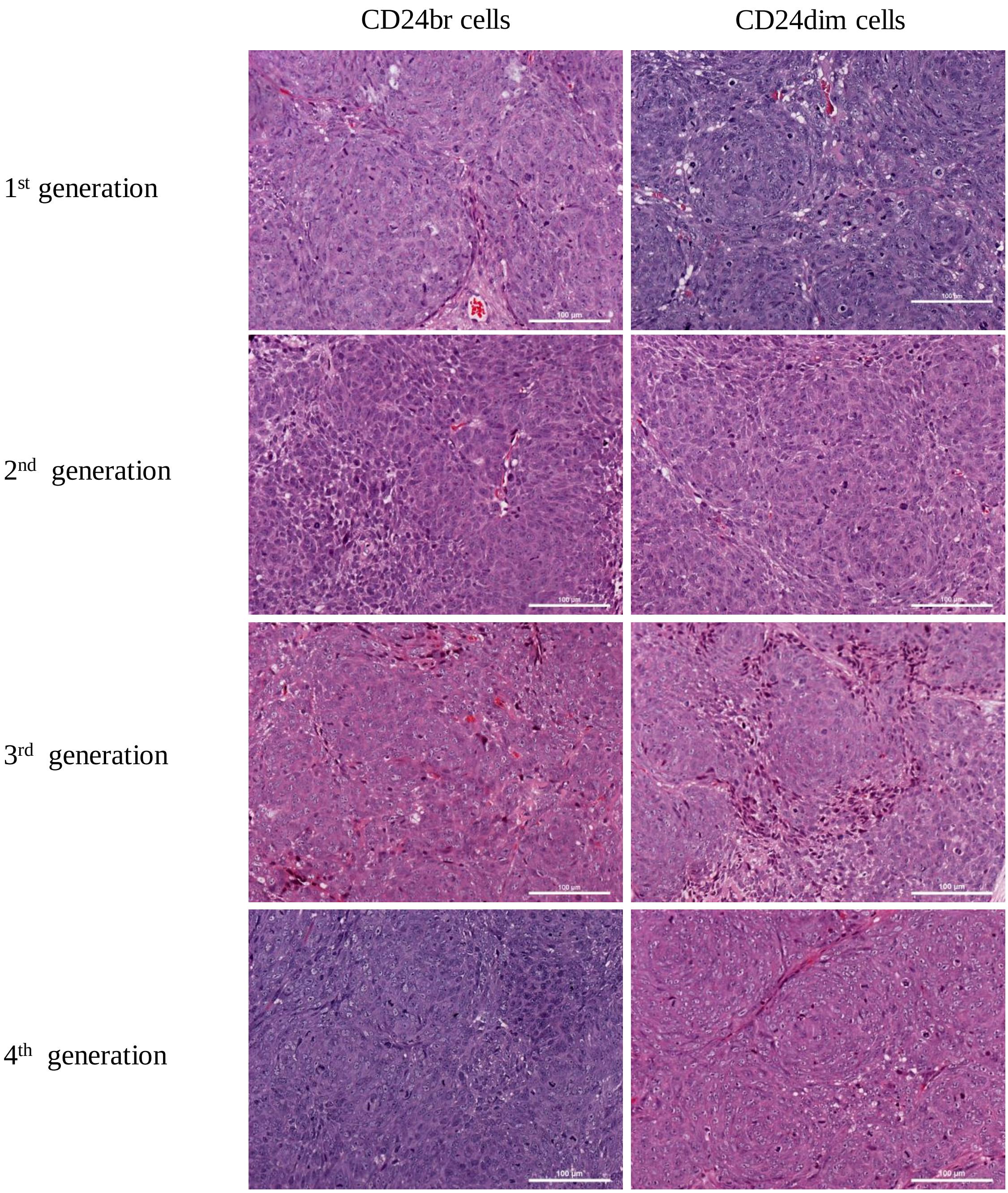

Figure S6 | Representative H&E images of resulting xenografts from xeno-B110 CD24br and CD24dim cells (1st generation) and subsequent serially-passaged generations. 20X objective; scale bar 100 µm.

Assay	TaqMan ID
HPRT1	Hs01003267_m1
RPL13A	Hs01926559_g1
UBC	Hs008 24723_m1
BMI1	Hs00180411_m1
CCND1	Hs00765553_m1
CCNE1	Hs01026536_m1
CD24	Hs02379687_s1
CD44 (all 8 isoforms)	Hs01075862_m1
CDKN1A	Hs00355782_m1
CTNNB1	Hs00355049_m1
EPCAM	Hs00158980_m1
KLF4	Hs00358836_m1
LMP1	custom
LMP2A	custom
MKI67	Hs01032443_m1
MYC	Hs00905030_m1
NANOG	Hs02387400_g1
NOTCH1	Hs01062014_m1
OCT4A	Hs03005111_g1
SOX2	Hs01053049_s1
VIM	Hs00185584_m1

Table S5 | TaqMan assays used in RT-qPCR.

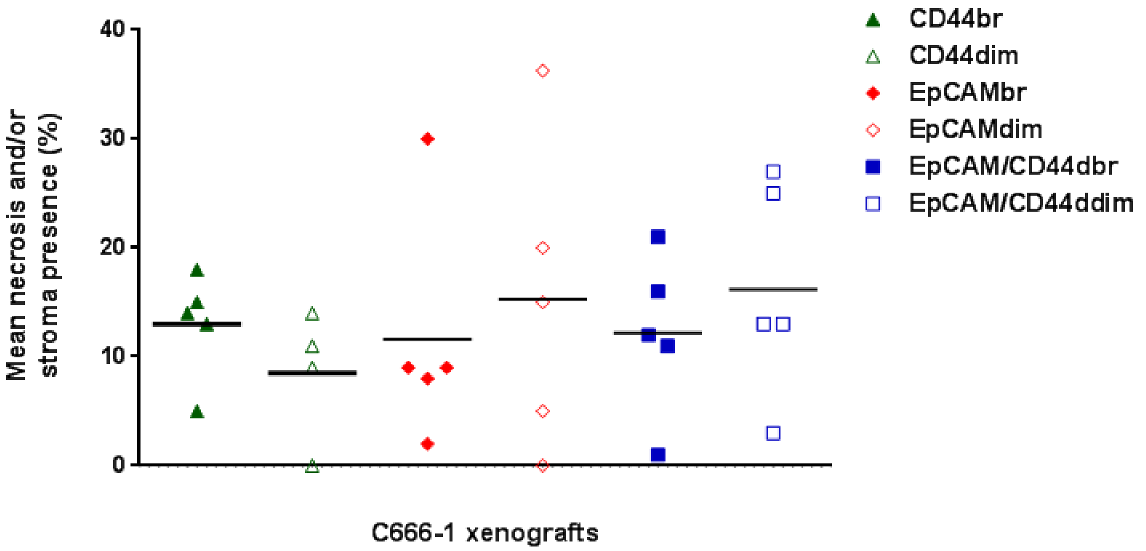

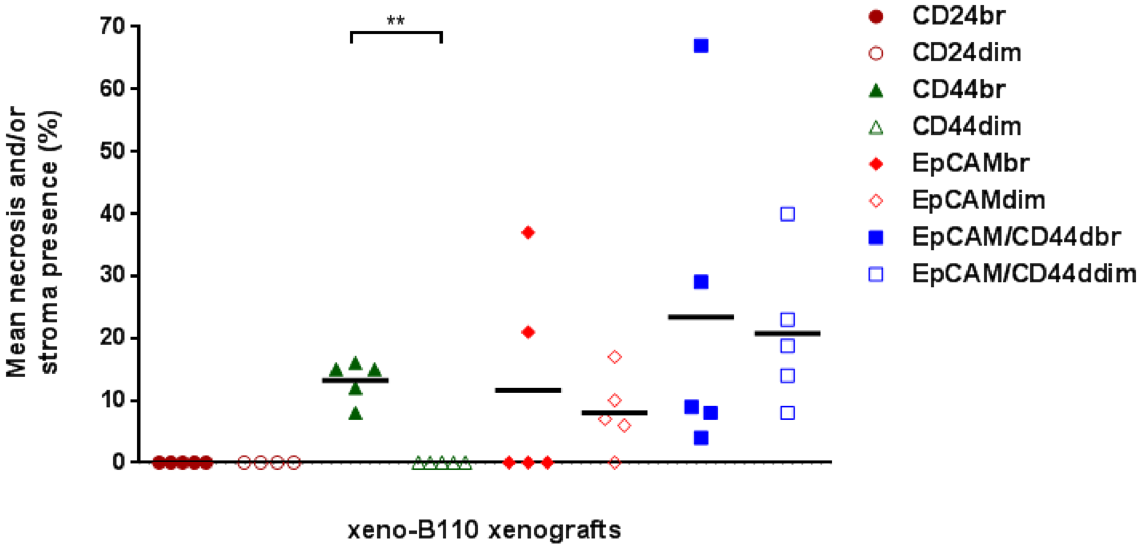

Figure S7 | The extent of necrosis and/or stroma in marker-selected cell-induced C666-1 and xeno-B110 xenografts. Each dot represents a xenograft tumour. **$p<0.001$

Cell phenotype			CD24 br	CD24 dim	CD44 br	CD44 dim	EpCAM br	EpCAM dim	EpCAM/ CD44dbr	EpCAM/ CD44ddim
KLF4	Pathologist 1	% positivity score	2	3	3	4	1	3	2	4
		staining intensity score	2	2	2	3	2	2	2	2
		total score	4	5	5	7	3	5	4	6
	Pathologist 2	% positivity score	3	4	3	4	2	3	2	3
		staining intensity score	2	3	2	3	1	2	1	2
		total score	5	7	5	7	3	5	3	5
	overall total score		4.5	6	5	7	3	5	3.5	5.5
p21	Pathologist 1	% positivity score	0	0	0	0	0	0	1	1
		staining intensity score	1	1	1	1	1	1	2	2
		total score	1	1	1	1	1	1	3	3
	Pathologist 2	% positivity score	0	0	1	1	0	0	0	1
		staining intensity score	1	2	2	3	2	1	3	2
		total score	1	2	3	4	2	1	3	3
	overall total score		1	1.5	2	2.5	1.5	1	3	3
cyclin D1	Pathologist 1	% positivity score	3	2	3	3	4	4	2	2
		staining intensity score	1	1	2	2	2	2	1	2
		total score	4	3	5	5	6	6	3	4
	Pathologist 2	% positivity score	2	2	2	2	2	3	4	3
		staining intensity score	1	1	1	1	1	2	2	3
		total score	3	3	3	3	3	5	6	6
	overall total score		3.5	3	4	4	4.5	5.5	4.5	5

Table S6 | Immunocytochemical analysis of KLF4, p21 and cyclin D1 expression in CD24, CD44 , EpCAM and EpCAM/CD44 groups of cells from xeno-B110.

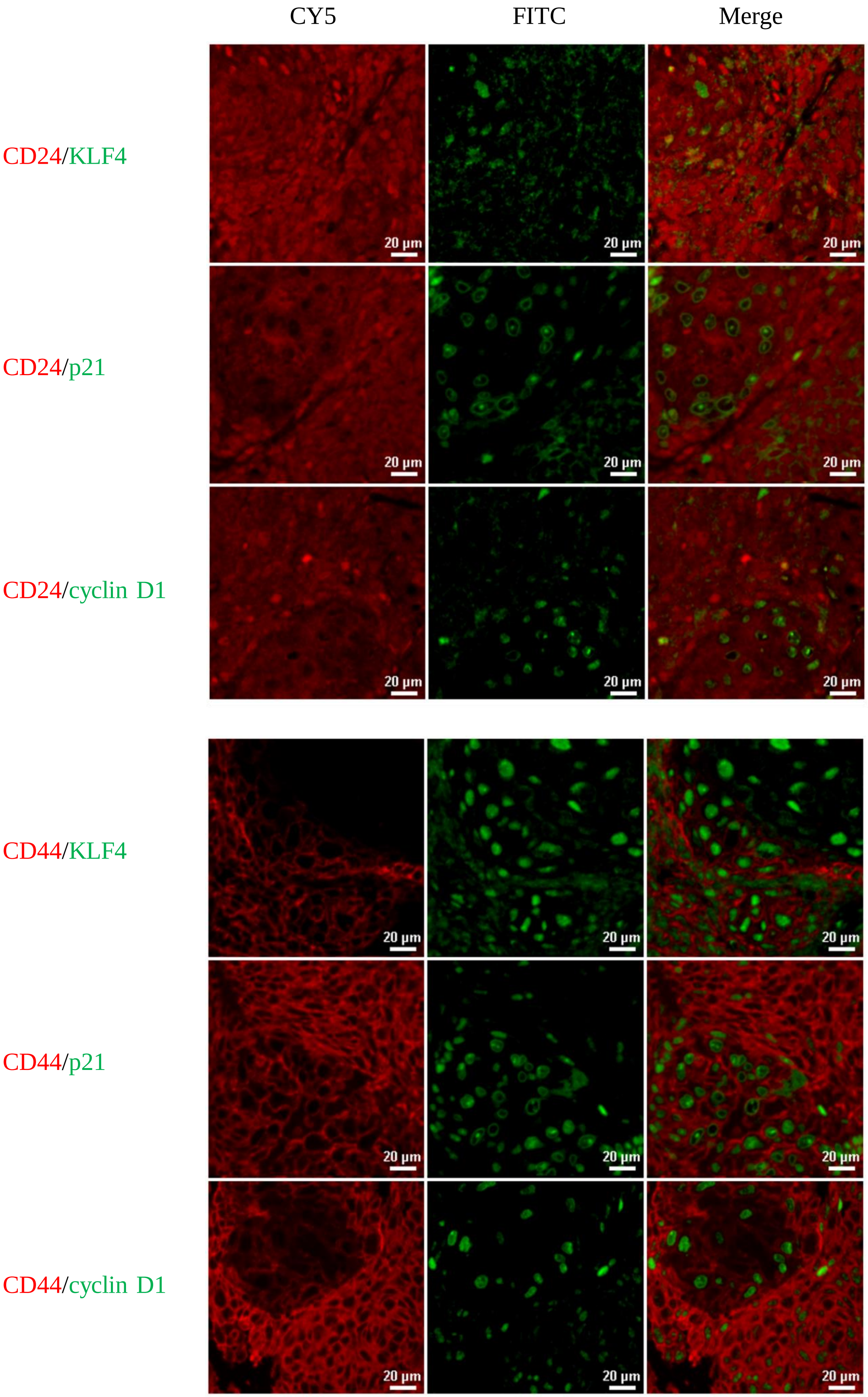

Figure S8 | Representative images of immunofluorescence (IF) analysis of CD24 and CD44 independently co-stained with KLF4, p21 and cyclin D1 in FFPE sections of xeno-B110. 20X objective; scale bar 100 μm.
